# Interplay of Affinity and Surface Tethering in Protein Recognition

**DOI:** 10.1021/acs.jpclett.2c00621

**Published:** 2022-04-29

**Authors:** Ali Imran, Brandon S. Moyer, Aaron J. Wolfe, Michael S. Cosgrove, Dmitrii E. Makarov, Liviu Movileanu

**Affiliations:** † Department of Physics, Syracuse University, 201 Physics Building, Syracuse, New York 13244-1130, United States; ‡ Ichor Life Sciences, Inc., 2651 US Route 11, LaFayette, New York 13084, United States; § Lewis School of Health Sciences, Clarkson University, 8 Clarkson Avenue, Potsdam, New York 13699, United States; ∥ Department of Chemistry, State University of New York College of Environmental Science and Forestry, 1 Forestry Dr., Syracuse, New York 13210, United States; ⊥ Department of Biochemistry and Molecular Biology, State University of New York Upstate Medical University, 4249 Weiskotten Hall, 766 Irving Avenue, Syracuse, New York 13210, United States; # Department of Chemistry, University of Texas at Austin, Austin, Texas 78712, United States; % Oden Institute for Computational Engineering and Sciences, University of Texas at Austin, Austin, Texas 78712, United States; & Department of Biomedical and Chemical Engineering, Syracuse University, 329 Link Hall, Syracuse, New York 13244, United States; $ The BioInspired Institute, Syracuse University, Syracuse, New York 13244, United States

## Abstract

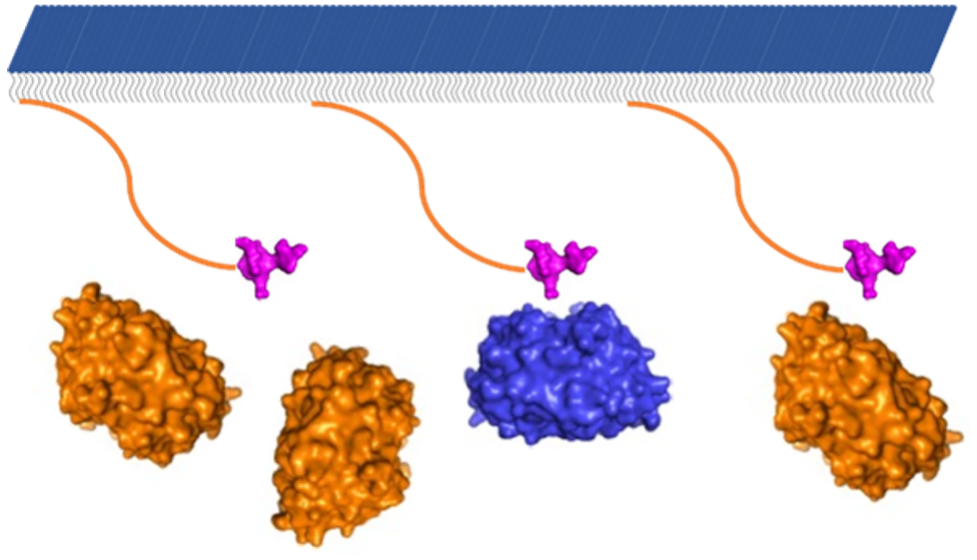

Surface-tethered ligand–receptor complexes are key components in biological signaling and adhesion. They also find increasing utility in single-molecule assays and biotechnological applications. Here, we study the real-time binding kinetics between various surface-immobilized peptide ligands and their unrestrained receptors. A long peptide tether increases the association of ligand–receptor complexes, experimentally proving the fly casting mechanism where the disorder accelerates protein recognition. On the other hand, a short peptide tether enhances the complex dissociation. Notably, the rate constants measured for the same receptor, but under different spatial constraints, are strongly correlated to one another. Furthermore, this correlation can be used to predict how surface tethering on a ligand–receptor complex alters its binding kinetics. Our results have immediate implications in the broad areas of biomolecular recognition, intrinsically disordered proteins, and biosensor technology.

Tethered ligand–receptor complexes are common in protein recognition^1,2^ and cellular adhesion.^3^ Surface-bound ligand–protein complexes are also the basis for biotechnological applications, such as biosensors^4−9^ and cell-targeted therapeutic proteins,^10,11^ as well as for single-molecule techniques that probe the dynamics and thermodynamics of protein binding.^12−16^ Yet, how the presence of spatial constraints imposed by the surface and/or the tether affects the thermodynamics and, especially, kinetics of binding is largely an open experimental question. Most of the current insight into this topic comes from theoretical^17−21^ and computational^10,22−24^ studies. However, experimental examinations of tethered ligand–protein interactions are mostly limited to measuring macroscopic intermolecular forces,^25−28^ equilibrium dissociation constants,^29^ and effective protein concentrations.^29,30^

In contrast to the earlier experimental work, this study focuses on the question of how the kinetics of binding and unbinding is altered by the tethering of one of the binding partners to a surface. To this end, we measure the real-time kinetics of tethered ligand–receptor complexes using surface immobilization-based sensing approaches. In our case, the receptor is WD40 repeat protein 5 (WDR5),^31,32^ a chromatin-associated hub that is primarily known for its regulatory role in histone methylation.^33,34^ The 334-residue WDR5 features a seven-bladed β propeller circular structure and a central cavity. The WDR5 cavity hosts the binding site for the WDR5-interaction (Win) motif of human mixed lineage leukemia (MLL/SET1) methyltransferases, also named the Win binding site. We examined details of the interactions of five 14-residue Win-motif peptide ligands of SET1 proteins (SET1_Win_ ligands; Table S1 and Supplemental Methods)^35,36^ with WDR5 via its Win binding site. SET1_Win_ ligands were chemically attached to a streptavidin-coated surface. Either a three-residue short peptide tether (ST-SET1_Win_ ligands; Figure 1a) or a nine-residue long peptide tether (LT-SET1_Win_ ligands; Figure 1b) was inserted between the biotinylated attachment site of the SET1_Win_ ligand to the surface and the SET1_Win_ sequence. In this way, the binding kinetics of the WDR5–SET1_Win_ complex was probed by using biolayer interferometry (BLI).^37^ The association and dissociation phases of the tethered ligand–receptor complex were discriminated optically by using changes in the interference pattern of reflected light waves at the sensor surface. Hence, these interactions were monitored by using WDR5-containing and WDR5-free assay buffers, respectively. Tethered ligand–receptor interactions were also evaluated by using Win binding site-directed WDR5 mutants (Table S2 and Supplemental Methods). To further examine the binding kinetics in the absence of restraining tethers, WDR5 proteins were immobilized on the surface plasmon resonance (SPR) sensors^38^ (no tether, NT-SET1_Win_ ligands; Figure 1c).

**Figure 1:**
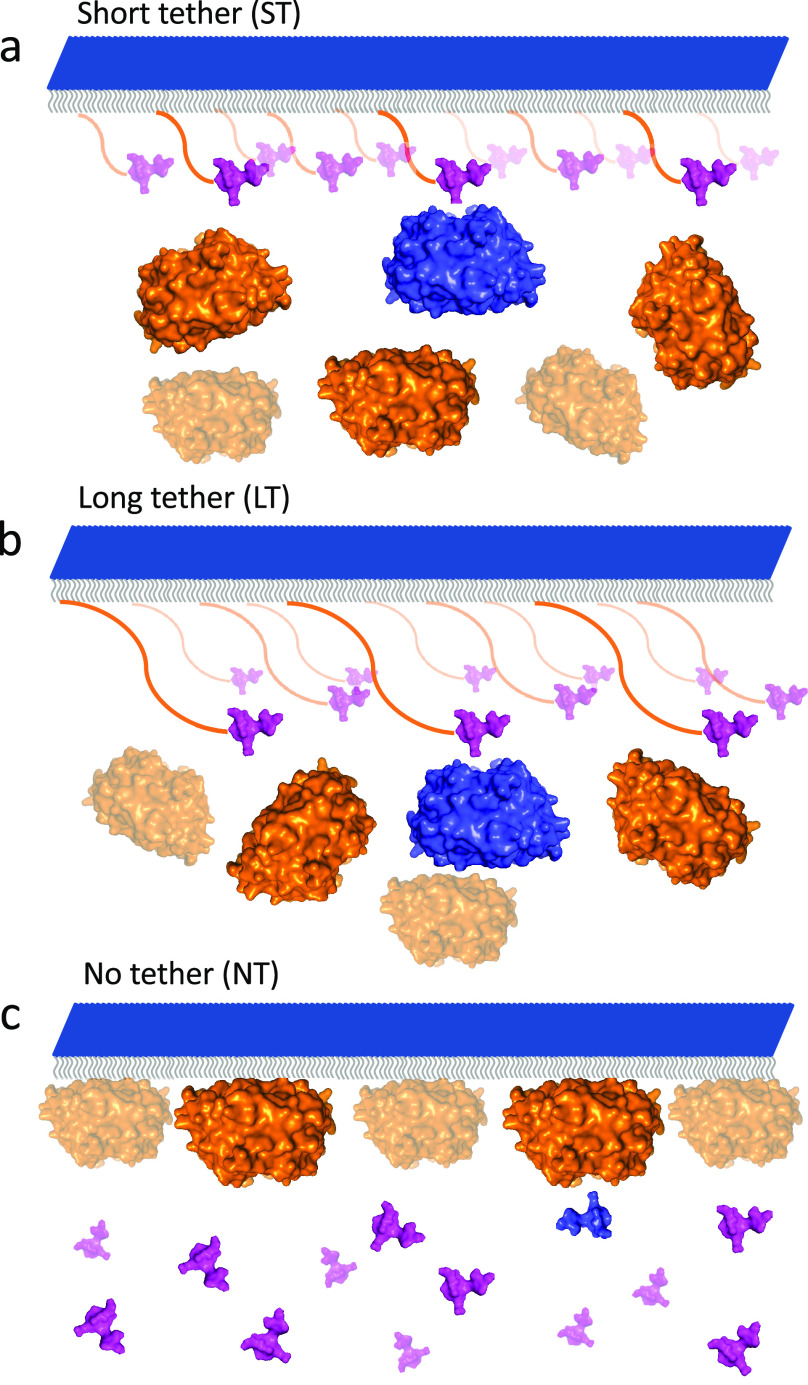
WDR5 protein interacting with the SET1_Win_ peptide ligands under different conditions. WDR5 is shown in orange, while SET1_Win_ ligands are shown in magenta. Bound interacting partners are shown in blue. Lightly colored receptors and ligands indicate interacting partners in the background. (a) Biotinylated ST-SET1_Win_ ligands were chemically attached onto a streptavidin-coated biolayer interferometry (BLI) sensor surface. Either WDR5 proteins or one of its mutants was freely movable in solution. (b) The same system as in (a), but with LT-SET1_Win_ ligands. (c) Either WDR5 proteins or one of its mutants was immobilized onto a surface plasmon resonance (SPR) chip surface, whereas the NT-SET1_Win_ ligands were freely movable in solution.

We obtained the real-time kinetics of five SET1_Win_ peptide ligands (MLL2_Win_, MLL3_Win_, MLL4_Win_, SETd1A_Win_, and SETd1B_Win_) with four WDR5 proteins (wild-type and 3 mutants of the Win binding site, P216L, F133L, and S218F) using ST and LT constraints (Figures S1 and S2,Tables S3–S5). Later, we validated the outcomes of this study using S175L, a fourth WDR5 mutant of unknown affinity. Interestingly, the association rate constants, *k*_a_, acquired with LT-SET1_Win_ ligands (*k*_a-LT_) were on average higher than those corresponding values recorded with ST-SET1_Win_ ligands (*k*_a-ST_) (Figure 2a and Table S6). To explain this observation, we considered the general framework of diffusion-controlled reactions,^39,40^ which gives the following association rate constant:
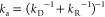
1where *k*_R_ is the reaction-controlled rate constant and
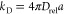
2is the diffusion-controlled rate constant that depends on the relative diffusion coefficient of the two reacting species, *D*_rel_, and on a “geometric” parameter, *a*. Here, *a* is the contact distance or capture radius between the centers of the two interacting partners considered as spheres. In the limit *k*_R_ ≫ *k*_D_, the association is purely diffusion controlled and *k*_a_ ≈ *k*_D_.^41^Equation 2 may be loosely interpreted as the rate constant of the association process happening instantaneously upon the reactants diffusing into a favorable relative configuration. This configuration is characterized by a linear length scale, *a*. Notably, simple dimensionality arguments require that the diffusion-controlled rate constant, *k*_D_, must be of the form of eq 2. Hence, eq 2 can be viewed as the definition of the *effective* “target” size of the diffusion-controlled reaction.

**Figure 2:**
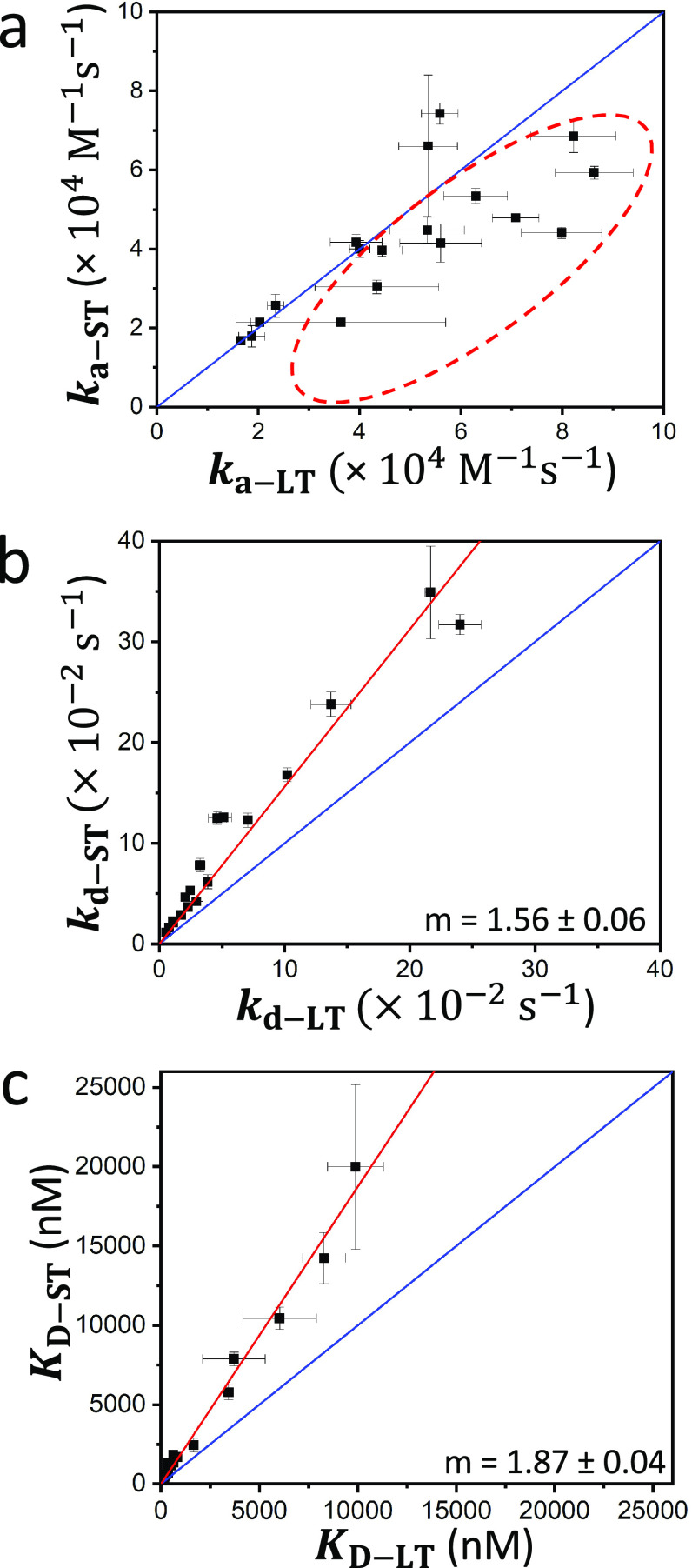
Scatter plots of kinetic and equilibrium constants for ST-SET1_Win_ and LT-SET1_Win_ ligands. (a) Association rate constants *k*_a-ST_ of ST-SET1_Win_-WDR5 complexes plotted against association rate constants *k*_a-LT_ of LT-SET1_Win_-WDR5 complexes. Points above the blue line correspond to complexes with faster association rate constants for ST-SET1_Win_ ligands, while points below correspond to interactions with slower association rate constants for ST-SET1_Win_ ligands. (b) Dissociation rate constants *k*_d-ST_ of ST-SET1_Win_–WDR5 complexes plotted against dissociation rate constants *k*_d-LT_ of LT-SET1_Win_–WDR5 complexes. Points above the blue line correspond to complexes with faster dissociation rate constants for ST-SET1_Win_ ligands. (c) Equilibrium dissociation constants *K*_D-ST_ of ST-SET1_Win_–WDR5 complexes plotted against equilibrium dissociation constants *K*_D-LT_ of LT-SET1_Win_–WDR5 complexes. Points above the blue line correspond to less stable complexes with ST-SET1_Win_ ligands. *m* indicates the slopes of linear fits in (b) and (c). Data represent mean ± s.d. which resulted from three independent BLI sensorgrams.

There are two notable examples of eq 2. First, Smoluchowski has obtained a formula for the diffusion-controlled rate constant, where the association process between two spherically symmetrical reactants takes place whenever their distance reaches the “capture radius” value *a*.^42,43^ Second, Berg and Purcell derived a formula for the rate constant of the process where a freely diffusing particle hits a patch on a planar wall, with *a* being the linear size of the patch.^44^ The Berg and Purcell scenario can be viewed as a prototype for the system studied here, as one of the reactants is surface immobilized.

It should be noted that the length parameter, *a*, generally depends on the interaction between the reactants.^39,40^ Therefore, the parameter *a* is not purely geometric.^45,46^ For example, for the model where the ligand and receptor are approximated as spheres interacting via a centrosymmetric potential, *U*(*r*), the diffusion-controlled rate constant to reach a geometric contact distance *R* is given by eq 2, with *a* defined as^39^
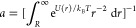
3where *k*_B_*T* is the thermal energy and *r* is the intersphere distance. Unless *U*(*r*) = 0, *a* is different from the pure geometric capture radius *R*. Consistent with intuition, for example, attractive electrostatic interaction increases the apparent value of *a*. Rotational diffusion and site-specific physical restrains of interacting molecules may further affect the apparent value of *a*. The sensitivity of the effective capture radius, *a*, to the interaction energy explains, at least in part, the small changes in the *k*_a_ between different SET1_Win_ peptide ligands (Figure 2a and Table S3).

Equipped with these ideas, we consider the difference between the cases of ST- and LT-SET1_Win_ ligands. The much smaller, surface-attached SET1_Win_ ligand diffuses rapidly, with a diffusion coefficient *D*_SET1Win_ ≫ *D*_WDR5_. Diffusion of the SET1_Win_ ligand occurs around its attachment point within a certain volume, which depends on the tether length. This suggests a simple model of association, as follows. Like in Berg and Purcell’s model,^44^ the surface-attached SET1_Win_ ligand appears as target with a characteristic size, *a*, to a freely diffusing WDR5. Because of the complicated geometry of the system, it is challenging to derive a simple expression for *a*. LT-SET1_Win_ can deviate further from the attachment point than ST-SET1_Win_. Therefore, LT-SET1_Win_ is a larger “target” for the WDR5 than ST-SET1_Win_ (i.e., *a*_LT_ > *a*_ST_), so the association rate constant for LT-SET1_Win_, *k*_a–LT_, is higher than that for ST-SET1_Win_, *k*_a–ST_, as observed in Figure 2a. Note, however, that this picture is expected to break down in the limit of long tethers where further increase of the tether length results in a larger search volume that has to be explored by the binding partners, reducing the overall association rate constant. Indeed, as recently discussed by Misiura and Kolomeisky,^45^ the dependence of the association rate constant on the tether length is nonmonotonic, with the maximum association speedup occurring at an intermediate tether length.

The association speedup induced by a longer tether found here is an experimental validation of the “fly-casting association mechanism”, which was proposed earlier by Wolynes and co-workers on theoretical grounds and computational analysis^47−49^ and discussed later by others.^13,45,50−54^ This mechanism explains how intrinsically disordered proteins with random-coil conformations can bind faster to their targets.^12,55^ Because of the geometric nature of the parameter *a*, it is expectable that the ratio of *a* values for LT-SET1_Win_ and ST-SET1_Win_, *a*_LT_/*a*_ST_, is nearly the same for all SET1_Win_ ligands. Indeed, we observe a linear correlation between the association rate constants for LT-SET1_Win_ and ST-SET1_Win_, *k*_a–LT_ and *k*_a–ST_, respectively (Figure 2a). But recalling that the parameter *a* also depends on the energetics of the interactions, deviations from a perfectly linear correlation are not surprising.

In contrast to the association rate constants, the dissociation rate constants for ST-SET1_Win_ ligands, *k*_d-ST_, were consistently higher than those for LT-SET1_Win_ ligands, *k*_d-LT_ (Figure 2b; Tables S7 and S8). Furthermore, *k*_d-ST_ and *k*_d-LT_ values closely followed a proportionality relationship. To explain these observations, we start with the Arrhenius law for the unimolecular dissociation process:^41^
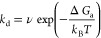
4where ν is a prefactor and Δ*G*_a_ is the activation free energy, which is determined by the strength of cohesive interactions between SET1_Win_ and WDR5. It is known that a microscopic object (e.g., a Brownian particle) tethered to a surface via a flexible polymer tether experiences a repulsive net force that pushes it away from the surface even when the surface is perfectly neutral. This is a typical situation in a single-molecule experiment, where a microscopic bead is anchored to a surface. This force is “entropic” in its nature, originating from the fact that the bead has more space available when it is further away from the surface. For example, if the tether length becomes very small, then this exclusion-volume effect is significant, resulting in a steric wall repulsion of the bead from the surface.^28^ The properties of this force have been theoretically studied by Segall and co-workers,^56^ who showed that it is roughly inversely proportional to the distance from the surface. Our real-time binding kinetics experiments with ST-SET1_Win_ involve a three-residue tether. This means that our ST-SET1_Win_ ligand–WDR5 receptor complex, whose size is ∼4.5 nm, is constrained to statistically fluctuate at a distance shorter than ∼1 nm from the surface. Here, we speculate that under these conditions the exclusion-volume effect of the tethered complex pushes WDR5 away from the surface and thus from SET1_Win_ as well. In this way, the steric wall repulsion enhances the dissociation of WDR5 receptors from SET1_Win_ ligands by lowering their dissociation barrier.

The simplest approximate description of this mechanochemical effect for the dissociation rate constant, *k*_d_, is the Eyring–Zhurkov–Bell formula:^57^
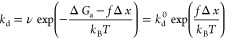
5where *k*_d_^0^ is *k*_d_ at *f* = 0. Here, *f* is the magnitude of the force, and Δ*x* is an activation length. Hence, *k*_d_^0^ is the dissociation rate constant in the absence of the surface. Clearly, the force *f* for ST-SET1_Win_, *f*_ST_, is higher than that for LT-SET1_Win_, *f*_LT_. Therefore, the dissociation rate constant for ST-SET1_Win_, *k*_d–ST_, is greater than that for LT-SET1_Win_, *k*_d–LT_, as observed in Figure 2b. Assuming that the activation length Δ*x*, being again a geometric parameter, is approximately the same for different constructs, the ratio of the two dissociation rate constants should be close to a constant. This should happen even though the rate constants themselves may vary considerably because of the variation of the activation free energy, Δ*G*_a_, and to exponential sensitivity of the dissociation rate constant to the energetics of interaction. Indeed, this is what we observe in Figure 2b. Despite almost 2 orders of magnitude variation between the individual *k*_d_ constants for each construct, *k*_d–ST_ and *k*_d–LT_ remain proportional to each other. Note that the *k*_a_ constants for the same constructs vary within a much narrower range, within a maximum factor of ∼4, supporting the above proposal that the association process is near the diffusion-controlled limit and thus less sensitive to energetics.

These results suggest that the length of the tether plays a significant role in modulating the interactions of the SET1_Win_–WDR5 complex. An increased physical constraint as a result of a decreased tether length not only reduces the rate constant of complex formation, as established earlier, but also substantially decreases the stability of the complex. Consequently, the overall impact of reducing the tether length is an increase in *K*_D_ (Figure 2c; Tables S9 and S10). Changes observed for *k*_a_ should normally be independent from those noted for *k*_d_ because the mechanisms of changing the corresponding activation free energies are different. Indeed, we observed no correlation between the *k*_a_ and *k*_d_ values (Figures S3 and S4).

We then measured the kinetic rate constants for 20 ligand–receptor complexes using unrestricted conditions (no tether, NT-SET1_Win_ ligands) (Figure S5, Tables S11–S13). In this case, BLI was not used because it does not have a satisfactory sensitivity to reliably detect a short-peptide binding to the surface. The SPR,^38^ with its greater sensitivity, was a more effective choice for this case. Accumulation of ligand–receptor complexes onto the surface of the SPR sensor was monitored by changes in the refractive index. Therefore, WDR5 was immobilized onto the surface of the SPR chips (Figure 1c), and the association and dissociation phases were probed in real time. As established by our previous work,^58^ the *k*_a_ values for NT-SET1_Win_ ligands were substantially greater than those for LT-SET1_Win_ ligands (Figure 3a and Table S14). This significant difference is due to the increased translational and rotational diffusion coefficients of NT-SET1_Win_ ligands relative to WDR5 and its derivatives. Moreover, our previous work^58^ also showed, by comparison with values obtained from fluorescence polarization (FP) spectroscopy, that immobilizing WDR5 onto the SPR sensor surface does not impact its functional integrity. Let us assume that *D*_NT–SET1Win_ and *D*_WDR5_ are the translational diffusion coefficients of NT-SET1_Win_ and WDR5, respectively. For applying eq 2 to this problem, one now has to consider that *D*_NT–SET1Win_ ≫ *D*_WDR5_ because either WDR5 or one of its derivatives was immobilized on the sensor surface. Therefore, the unrestrained NT-SET1_Win_ was responsible for the diffusion-mediated mutual approach of the reacting species, so *D*_rel_ ≈ *D*_NT–SET1Win_. Again, eq 2 predicts proportionality between *k*_a–NT_ and *k*_a–LT_, as noted in Figure 3a, with the ratio of the two roughly equal to the ratio of SET1_Win_’s and WDR5’s diffusion coefficients.

**Figure 3:**
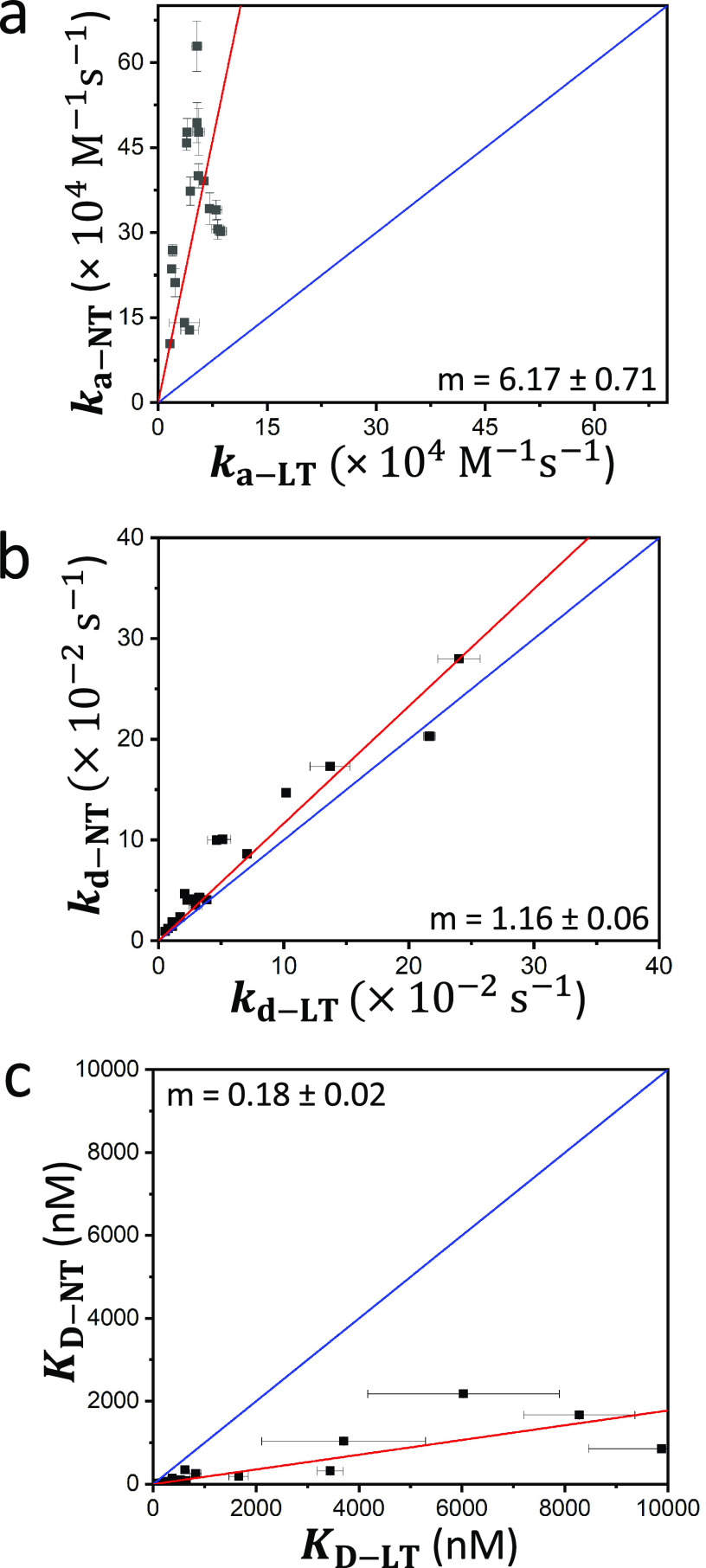
Scatter plots of kinetic and equilibrium constants for NT-SET1_Win_ and LT-SET1_Win_ ligands. (a) Association rate constants *k*_a-NT_ of NT-SET1_Win_–WDR5 complexes plotted against association rate constants *k*_a-LT_ of LT-SET1_Win_–WDR5 complexes. Points above the blue line correspond to interactions with faster association rate constants for NT-SET1_Win_ ligands. (b) Dissociation rate constants *k*_d-NT_ of NT-SET1_Win_–WDR5 complexes plotted against dissociation rate constants *k*_d-LT_ of LT-SET1_Win_–WDR5 complexes. Points above the blue line correspond to interactions with faster dissociation rate constants for NT-SET1_Win_ ligands. (c) Equilibrium dissociation constants *K*_D-NT_ values of NT-SET1_Win_–WDR5 complexes plotted against equilibrium dissociation constants *K*_D-LT_ of LT-SET1_Win_–WDR5 complexes. Points below the blue line correspond to more stable complexes with LT-SET1_Win_ ligands. *m* indicates the slopes of linear fits in all panels. Data represent mean ± s.d. which resulted from three independent BLI sensorgrams.

Remarkably, the *k*_d_ values using NT-SET1_Win_ and LT-SET1_Win_ ligands were closely similar (Figure 3b and Table S15). Our interpretation of this finding is in terms of eq 5. In the case of LT-SET1_Win_ ligands, but not for ST-SET1_Win_ ligands, the repulsive force *f* is negligible as the complex is far enough from the surface. Hence, the dissociation rate constant is near that value corresponding to the zero-force limit, *k*_d_^0^, which is the dissociation rate constant for NT-SET1_Win_ ligands, *k*_d–NT_. In other words, at long enough tether lengths, the experimental system approaches that of NT-SET1_Win_ ligands in terms of the dissociation rate constant, *k*_d_. Therefore, the equilibrium dissociation constant, *K*_D_, of the ligand–receptor complex becomes larger as we go from NT-SET1_Win_ ligands to LT-SET1_Win_ ligands (Figure 3c; Tables S16 and S17). Moreover, the differential free energy of the ligand–receptor complex formation, ΔΔ*G*, for NT-SET1_Win_ ligands with respect to LT-SET1_Win_ ligands is in the range −0.3 through −1.5 kcal/mol. The primary contribution to this change results from the considerable increase in the *k*_a_ in the absence of the tether. This shows how the attachment of a binding partner to a surface influences the overall dynamic equilibrium of the interaction. In our case, the effect is substantial given the large difference in size between the two binding partners. Even though for NT-SET1_Win_ ligands the WDR5 is restricted to the surface, the comparison between similar restriction and steady-state fluorescence polarization (FP) data of freely interacting SET1_Win_ and WDR5 in solution shows that this condition can be thought as that of an unrestricted interaction.^58^

In Figure 4a, we illustrate a qualitative comparison of the free energy landscapes that correspond to NT-SET1_Win_, ST-SET1_Win_, and LT-SET1_Win_ ligands. For short and long tethers, the presence of the flexible tether reduces the association rate constant of the SET1_Win_–WDR5 complex with respect to that in the absence of the tether (Figure S6). Further increase in the *k*_d-ST_ with respect to *k*_d-LT_ (Figure S7) due to repulsion forces of WDR5 proteins from the sensor surface explains the relative increase in the normalized values (*K*_D-ST_**/***K*_D-NT_) > (*K*_D-LT_**/***K*_D-NT_) (Figure 4b–e). Because there are linear correlations between measured affinities of various SET1_Win_–WDR5 pairs with specified constraints, we can advantageously utilize these findings to predict the *k*_d_ and *K*_D_ for a given tethered ligand–receptor complex. To demonstrate this, we examined the interactions of SET1_Win_ ligands with S175L, a WDR5 derivative, whose single-site mutation is located within the Win binding site. Using the kinetic and equilibrium parameters measured for NT-SET1_Win_–S175L interactions via SPR (Tables S12 and S13), we established the proportionality relationships with their corresponding parameters for ST-SET1_Win_ ligands (Figure S8). Remarkably, our experimental determinations of *k*_d-ST_ for S175L against five ST-SET1_Win_ ligands are closely similar to corresponding anticipated values (Table 1). Furthermore, using the same method, we demonstrate the predictive power of this approach for the *K*_D-ST_ values (Table 2). Therefore, the binding affinity of tethered ligand–receptor interactions can be precisely modulated by changing the tether length (Figure S9).

**Table 1:** Predicted and Experimental Values of the *k*_d-ST_ for S175L Interacting with ST-SET1_Win_^a^

parameter	SET1_Win_	predicted values ×10^3^ (s^–1^)	experimental values ×10^3^ (s^–1^)
*k*_d-ST_	MLL2_Win_	14 ± 1	12 ± 1
	MLL3_Win_	36 ± 1	28 ± 1
	MLL4_Win_	190 ± 10	180 ± 10
	SETd1A_Win_	300 ± 10	160 ± 10
	SETd1B_Win_	13 ± 1	5.4 ± 0.2

a *k*_d-ST_ are the dissociation rate constants corresponding to ST-SET1_Win_ ligands. Predicted values of *k*_d-ST_ were obtained using the proportionality relationship between *k*_d-ST_ and *k*_d-NT_ (Figure S8) and the experimentally determined values of *k*_d-NT_ (Table S12). Triplicate *k*_d-NT_ values were used to calculate corresponding *k*_d-ST_ values by linear interpolation. Values indicate mean ± s.d., which were calculated by using these triplicates.

**Table 2:** Predicted and Experimental Values of the *K*_D-ST_ for S175L Interacting with ST-SET1_Win_^a^

parameter	SET1_Win_	predicted values ×10^9^ (M)	experimental values ×10^9^ (M)
*K*_D-ST_	MLL2_Win_	150 ± 10	360 ± 30
	MLL3_Win_	270 ± 10	810 ± 90
	MLL4_Win_	2800 ± 100	8500 ± 300
	SETd1A_Win_	5500 ± 200	2900 ± 100
	SETd1B_Win_	110 ± 10	110 ± 6

a *K*_D-ST_ are the equilibrium dissociation constants corresponding to ST-SET1_Win_ ligands. Predicted values of *K*_D-ST_ were obtained using the proportionality relationship between *K*_D-ST_ and *K*_D-NT_ (Figure S8) and the experimentally determined values of *K*_D-NT_ (Table S13). Triplicate *K*_D-NT_ values were used to calculate corresponding *K*_D-ST_ values by linear interpolation. Values indicate mean ± s.d., which were calculated by using these triplicates.

**Figure 4:**
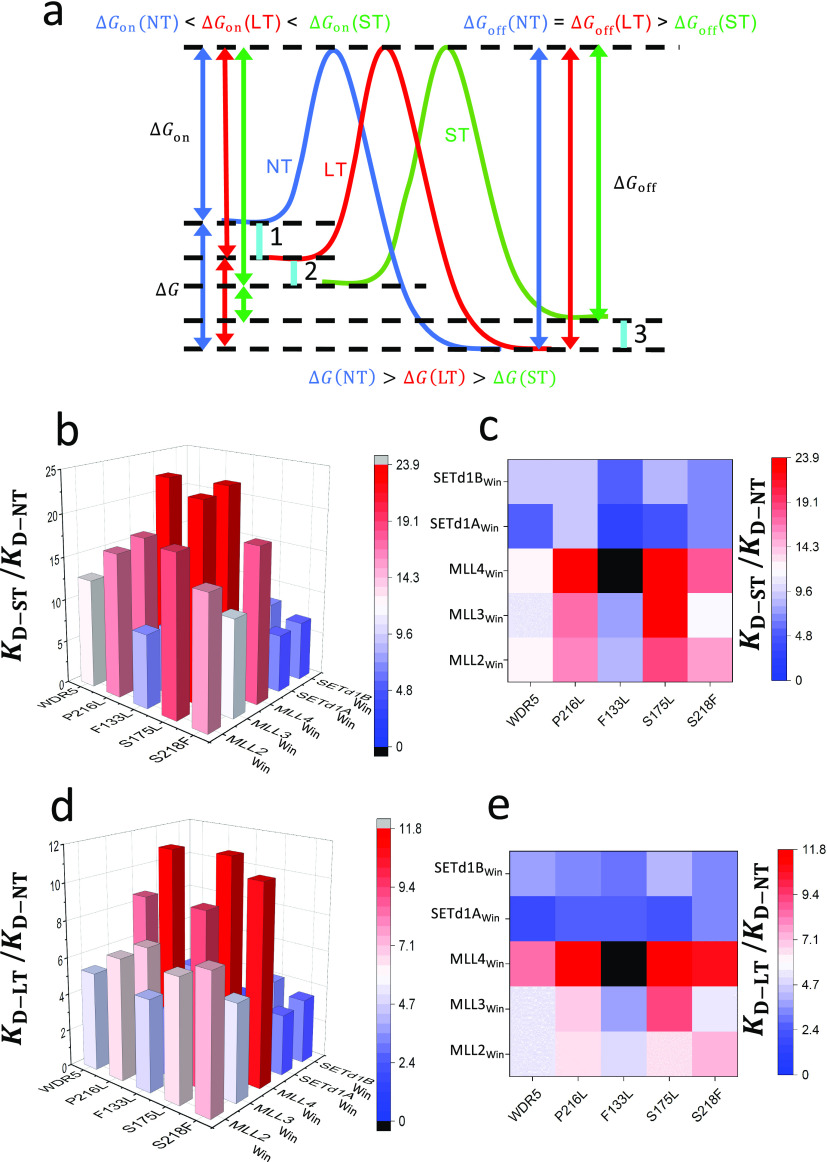
3D plots and contour maps of normalized *K*_D_ constants. (a) Qualitative free energy landscapes of SET1_Win_–WDR5 interactions when NT-SET1_Win_ (NT), ST-SET1_Win_ (ST), and LT-SET1_Win_ (LT) peptide ligands were used. Vertical lines 1, 2, and 3, which are marked in cyan, indicate the differential free energy barriers due to unrestrained diffusion of the ligand, fly casting mechanism, and repulsion entropic forces of the receptor from the sensor surface, respectively. (b) Bar graph and (c) contour map of *K*_D-ST_ values for the interaction of ST-SET1_Win_ ligands, with WDR5 and its mutants, divided by their corresponding *K*_D-NT_ values measured with the corresponding NT-SET1_Win_ ligands. (d) Bar graph and (e) contour map of *K*_D-LT_ values for the interaction of LT-SET1_Win_ ligands, with WDR5 and its mutants, divided by their corresponding *K*_D-NT_ values measured with the corresponding NT-SET1_Win_ ligands. *K*_D-ST_ and *K*_D-LT_ for MLL4_Win_–F133L interactions could not be quantitatively determined by using BLI measurements. These data points are colored in black.

In summary, we provide compelling experimental evidence for the fly casting mechanism of association between surface-attached peptide ligands and their receptors. The observed speedup in the association rate, *k*_a_, when using a longer tether is rather modest for the tether lengths employed here, which agrees with previous computational work.^47^ We also found that the dissociation rate constant, *k*_d_, was greater in the case of a short tether length as a result of steric wall repulsion forces acting on the receptor pulling it away from the surface. Accordingly, this resulted in a weakened interaction of the tethered ligand–protein complex. As a longer tether accelerates the association but decelerates the dissociation, the binding affinity of the ligand–receptor complex is greater at increased tether lengths. From a practical point of view, our experimental approach can be used to predict dissociation rate constants and binding affinities of ligand–protein interactions for specified physicochemical properties of the tether. This study also reveals that the surface immobilization-based experiments are expected to provide different kinetic and equilibrium fingerprints of the tethered ligand–receptor interactions with respect to unrestrained conditions. For example, we show that the association rate constants of ligand–receptor interactions under NT conditions are about 1 order of magnitude greater than those acquired under LT conditions. In addition, we anticipate that the nature of the linker might impact these parameters as well. Therefore, our method can be employed in biosensor technology to modulate the interaction strength of a ligand–protein complex on a sensing surface by modifying the tether length. Finally, this result has been successfully validated by using a test WDR5 mutant of unknown dissociation constant for five ST-SET1_Win_ ligands.

## Supporting Information Available

Peptide synthesis, purification, and analysis, protein expression and purification, biolayer interferometry, surface plasmon resonance, examples of BLI sensorgrams, determinations of the kinetic and equilibrium constants, scatter plots of the association rate constants versus the dissociation rate constants, examples of SPR sensorgrams, 3D plots and contour maps of the association and dissociation rate constants, supporting references (PDF)
