# Supplementary material for: Interplay of Affinity and Surface Tethering in Protein Recognition
Source: J Phys Chem Lett. 2022 Apr 29;13(18):4021–8. doi: 10.1021/acs.jpclett.2c00621 (PMC9106920; doi:10.1021/acs.jpclett.2c00621)
Supplement: Supplementary file 1 — jz2c00621_si_001.pdf [file jz2c00621_si_001.pdf]

SUPPLEMENTARY INFORMATION FILE

Interplay of Affinity and Surface Tethering in Protein Recognition

Ali Imran,[1] Brandon S. Moyer,[2] Aaron J. Wolfe,[1,2,3,4] Michael S. Cosgrove,[5] Dmitrii E. Makarov,[6,7] and Liviu Movileanu[1,8,9,*]

[1]*Department of Physics, Syracuse University, 201 Physics Building, Syracuse, New York 13244-1130, USA*

[2]*Ichor Life Sciences, Inc., 2651 US Route 11, LaFayette, New York 13084, USA*

[3]*Department of Chemistry, State University of New York College of Environmental Science and Forestry, 1 Forestry Dr., Syracuse, New York 13210, USA*

[4]*Lewis School of Health Sciences, Clarkson University, 8 Clarkson Avenue, Potsdam, New York 13699*

[5]*Department of Biochemistry and Molecular Biology, State University of New York Upstate Medical University, 4249 Weiskotten Hall, 766 Irving Avenue, Syracuse, New York 13210, USA*

[6]*Department of Chemistry, University of Texas at Austin, Austin, Texas 78712, USA*

[7]*Oden Institute for Computational Engineering and Sciences, University of Texas at Austin, Austin, Texas 78712, USA.*

[8]*Department of Biomedical and Chemical Engineering, Syracuse University, 329 Link Hall, Syracuse, New York 13244, USA*

[9]*The BioInspired Institute, Syracuse University, Syracuse, New York 13244, USA*

Keywords: *Fly-casting mechanism; Protein-peptide interactions; Biolayer interferometry; Kinetics; Peptide ligand; Surface plasmon resonance*

*The corresponding author's contact information:
Liviu Movileanu, PhD, Department of Physics, Syracuse University, 201 Physics Building, Syracuse, New York 13244-1130, USA. Phone: 315-443-8078; E-mail: lmovilea@syr.edu

Table of Contents (TOC) - Supplementary Information file

1. Materials and Methods (**Supplementary Tables S1-S2**).
1.1. Peptide synthesis, purification, and analysis
1.2. Protein expression and purification.
1.3. Biolayer interferometry (BLI).
1.4. Surface plasmon resonance (SPR).

2. Examples of BLI sensorgrams for probing real-time kinetics of $SET1_{Win}$-WDR5 interactions **(Supplementary Figs. S1-S2)**.

3. Determinations of the kinetic and equilibrium constants of the interactions of ST-$SET1_{Win}$ ligands with WDR5 receptors using BLI measurements (**Supplementary Tables S3-S5**).

4. The kinetic and equilibrium constants of the interactions of ST-$SET1_{Win}$ ligands with WDR5 receptors normalized to the corresponding values of LT-$SET1_{Win}$ ligands (**Supplementary Tables S6-S10**).

5. Scatter plots of the association rate constants versus the dissociation rate constants using linear- and logarithm-scale representations **(Supplementary Figs. S3-S4)**.

6. Examples of SPR sensorgrams for probing real-time kinetics of NT-$SET1_{Win}$-WDR5 interactions **(Supplementary Fig. S5)**.

7. Determinations of the kinetic and equilibrium constants of the interactions of NT-$SET1_{Win}$ ligands with WDR5 receptors using SPR measurements (**Supplementary Tables S11-S13**).

8. The kinetic and equilibrium constants of the interactions of NT-$SET1_{Win}$ ligands with WDR5 receptors normalized to the corresponding values of LT-$SET1_{Win}$ ligands (**Supplementary Tables S14-S17**).

9. The 3D plots and contour maps of the association rate constants under ST and LT conditions normalized to those recorded under NT conditions **(Supplementary Fig. S6)**.

10. The 3D plots and contour maps of the dissociation rate constants under ST and LT conditions normalized to those recorded under NT conditions **(Supplementary Fig. S7)**.

11. Scatter plots of kinetic and equilibrium constants for the ST, LT, and NT experiments **(Supplementary Figs. S8-S9)**.

12. Supporting references.

1. Materials and Methods

1.1. Peptide synthesis, purification, and analysis. For BLI measurements, 14-residue SET1$_{Win}$ peptide ligands were synthesized and purified to ≥ 95% purity by GenScript (Piscataway, NJ). These peptide ligands were biotinylated at their N terminus and amidated at their C terminus. Their sequence is provided in **Table S1**, which is displayed below.

Supplementary Table S1. List of 14-residue SET1$_{Win}$ peptide ligands used in biolayer interferometry (BLI) measurements. An either a 3-residue short tether (ST) or a 9-residue long tether (LT) was inserted between the biotinylated attachment site and SET1$_{Win}$ sequence. The tether sequence is marked in blue. The SET1$_{Win}$ sequence is marked in red.

#	SET1$_{Win}$	Tether length	Ligand sequence
1	ST-MLL2$_{Win}$	ST	Biotinyl-(GGS)INPTGCARSEPKIL$_{NH2}$
2	ST-MLL3$_{Win}$	ST	Biotinyl-(GGS)VNPTGCARSEPKMS$_{NH2}$
3	ST-MLL4$_{Win}$	ST	Biotinyl-(GGS)LNPHGAARAEVYLS$_{NH2}$
4	ST-SETd1A$_{Win}$	ST	Biotinyl-(GGS)EHQTGSARSEGYYP$_{NH2}$
5	ST-SETd1B$_{Win}$	ST	Biotinyl-(GGS)EHVTGCARSEGFYT$_{NH2}$
6	LT-MLL2$_{Win}$	LT	Biotinyl-(GGS)$_3$INPTGCARSEPKIL$_{NH2}$
7	LT-MLL3$_{Win}$	LT	Biotinyl-(GGS)$_3$VNPTGCARSEPKMS$_{NH2}$
8	LT-MLL4$_{Win}$	LT	Biotinyl-(GGS)$_3$LNPHGAARAEVYLS$_{NH2}$
9	LT-SETd1A$_{Win}$	LT	Biotinyl-(GGS)$_3$EHQTGSARSEGYYP$_{NH2}$
10	LT-SETd1B$_{Win}$	LT	Biotinyl-(GGS)$_3$EHVTGCARSEGFYT$_{NH2}$

Purity confirmation, amino acid analysis, and solubility testing were conducted and provided by GenScript. For SPR measurements, SET1$_{Win}$ peptide ligands were synthesized, purified, and analyzed in-house at Ichor Life Sciences (LaFayette, NY). Details on these procedures and protocols were previously provided [1]. Peptide synthesis was performed using a Biotage Syro I peptide synthesizer (Biotage, Charlotte, NC). Peptide purification was achieved using reversed-phase chromatography in two steps: (1) flash chromatography employing a Biotage Isolera One (Biotage AB, Uppsala, Sweden), and (2) semi-preparative high-performance liquid chromatography (HPLC) using a Waters 2695 separations module, which was coupled with a Waters 2996 photodiode array detector (PDA).

1.2. Protein expression and purification. In this study, all expression plasmids were synthesized, codon optimized, and sequence verified by GenScript (Piscataway, NJ). Human WDR5 (UniProtKB - P61964; WDR5_HUMAN) and its mutants were expressed and purified as described previously.[1-3] WDR5 construct design has the following sequence:
6H-TEV-**WDR5** in pET3aTr vector (Addgene, Watertown, MA). The detailed WDR5 sequence (sequence fragments marked in yellow are linkers) is the following:
MHHHHHHSSGVDLGTENLYFQSNG**ATEEKKPETEAARAQPTPSSSATQSKPTPVKPN
YALKFTLAGHTKAVSSVKFSPNGEWLASSSADKLIKIWGAYDGKFEKTISGHKLGIS
DVAWSSDSNLLVSASDDKTLKIWDVSSGKCLKTLKGHSNYVFCCNFNPQSNLIVSG
SFDESVRIWDVKTGKCLKTLPAHSDPVSAVHFNRDGSLIVSSSYDGLCRIWDTASGQ
CLKTLIDDDNPPVSFVKFSPNGKYILAATLDNTLKLWDYSKGKCLKTYTGHKNEK
YCIFANFSVTGGKWIVSGSEDNLVYIWNLQTKEIVQKLQGHTDVVISTACHPTENII
ASAALENDKTIKLWKSDC**

Supplementary Table S2. This table shows WDR5 mutants used in this study. These WDR5 mutants involve amino acid side chains within the Win binding site of WDR5.[4-6]

Entry	WDR5 Mutant
1	P216L
2	F133L
3	S218F
4	S175L

1.3. Biolayer interferometry (BLI). These measurements were conducted using an Octet RED384 instrument (FortéBio, Fremont, CA) at 24ºC.[7] The assay buffer included 150 mM NaCl, 20 mM Tris-HCl, 1 mM TCEP, 1 mg/mL bovine serum albumin (BSA), pH 7.5. Streptavidin-coated biosensors were incubated with 5 nM biotinylated $SET1_{Win}$ for 15 minutes. Then, the unbound peptides were washed out by rinsing the sensors in assay buffer. These experimental conditions were optimized to amplify the signal-to-noise ratio while preventing potential artifacts. These include the rebinding of receptors to the surface-immobilized peptide ligands during the dissociation phase. Prior crystallographic studies demonstrated that these ligand-receptor interactions follow a 1:1 binding model.[8,9] The association process was monitored by exposing the sensors to 3-fold serial dilutions of WDR5 proteins. The dissociation phase was probed by transferring the sensors into WDR5-free assay buffer. The association phases were fitted using the equation:[10]

$$Y = Y_\infty - (Y_\infty - Y_0)e^{-k_{\text{obs}}t} \quad \text{(S1)}$$

Here, Y_0 and Y_∞ denote the responses at the initial time and infinity, respectively. k_{obs} is the apparent first-order reaction rate constant of the association phase. t represents the cumulative time of the association reaction. The dissociation phases were fitted using the equation:

$$Y = Y_\infty + (Y_0 - Y_\infty)e^{-k_{\text{off}}t} \quad \text{(S2)}$$

Here, k_{off} indicates the dissociation rate constant. Y_0 and Y_∞ are the responses at the initial time and infinity, respectively. Finally, the association rate constant, k_{on}, was determined using the slope of the linear curve:[11,12]

$$k_{\text{obs}} = k_{\text{on}}[C] + k_{\text{off}} \quad \text{(S3)}$$

Then, global fittings were achieved using several WDR5 (or WDR5 mutant) concentrations. These fittings provided the corresponding k_{on} and k_{off} values. Equilibrium dissociation constant values, K_D, were indirectly determined using the k_{on} and k_{off} values ($K_D = k_{\text{off}}/k_{\text{on}}$). Three independent BLI measurements were conducted for all conditions in this study.

1.4. Surface plasmon resonance (SPR). In this study, all SPR measurements were conducted using a Cytiva Biacore 8K instrument (Cytiva Life Sciences, Marlborough, MA), as previously reported.[1] WDR5 proteins were immobilized onto the active flow cell of each channel of a Cytiva Series S Sensor Chip CM5 (Cytiva Life Sciences). The sensor surface was then activated using an injection of 1:1 N-hydroxysuccinimide (NHS)/1-ethyl-3-(3-dimethylaminopropyl)carbodiimide (EDC) (Cytiva Amine Coupling Kit, Cytiva Life Sciences). The protein sample was then injected across the active flow cell. Finally, both active and passive flow cells were chemically deactivated. Multicycle kinetic analyses were conducted at a flow cell temperature of 25°C and a sample compartment temperature of 20°C in a running buffer composed of 20 mM Tris-HCl (pH 7.5), 150 mM NaCl, 1 mM TCEP, 0.05% Tween 20. Biacore™ Insight Evaluation Software v3 (Cytiva Life Sciences) was employed to analyze and

fit the sensorgrams using a 1:1 binding interaction model to provide the association (k_a) and dissociation (k_d) rate constants. The K_D were calculated indirectly using $K_D = k_d/k_a$.

2. Examples of BLI sensorgrams and fittings for probing the real-time kinetics of SET1$_{Win}$-WDR5 interactions.

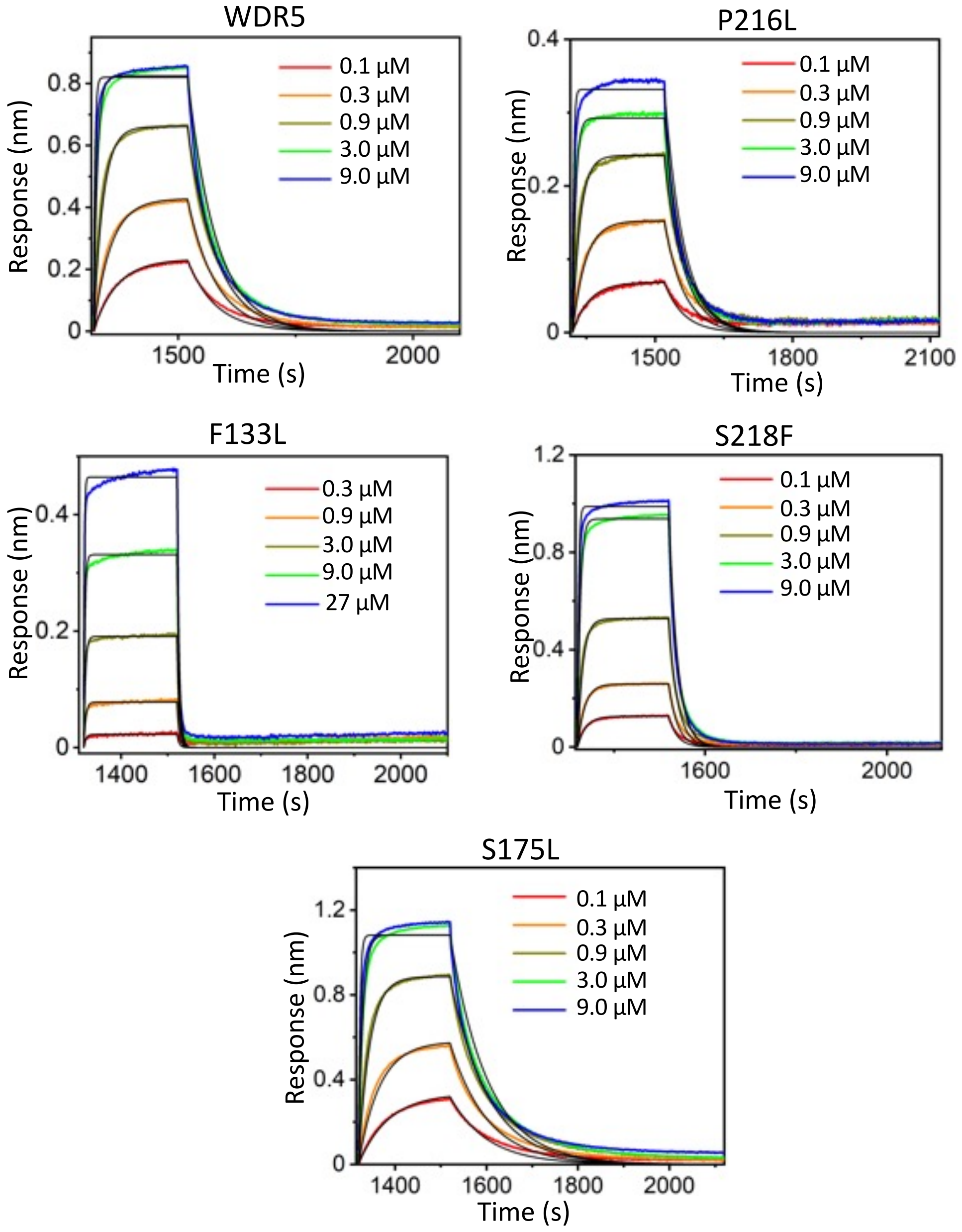

__Supplementary Fig. S1.__ **BLI sensorgrams of ST-MLL2$_{Win}$ interacting with WDR5 and its mutants.** 5 nM biotinylated ST-MLL2$_{Win}$ was loaded onto streptavidin-coated sensors for 15 minutes. 3-fold serial dilutions of WDR5 and its mutants were used to obtain individual binding curves. These sensorgrams were fitted to obtain $k_{a\text{-ST}}$, $k_{d\text{-ST}}$, and $K_{D\text{-ST}}$ (**eqs. S1-S3**). The fits are shown in black.

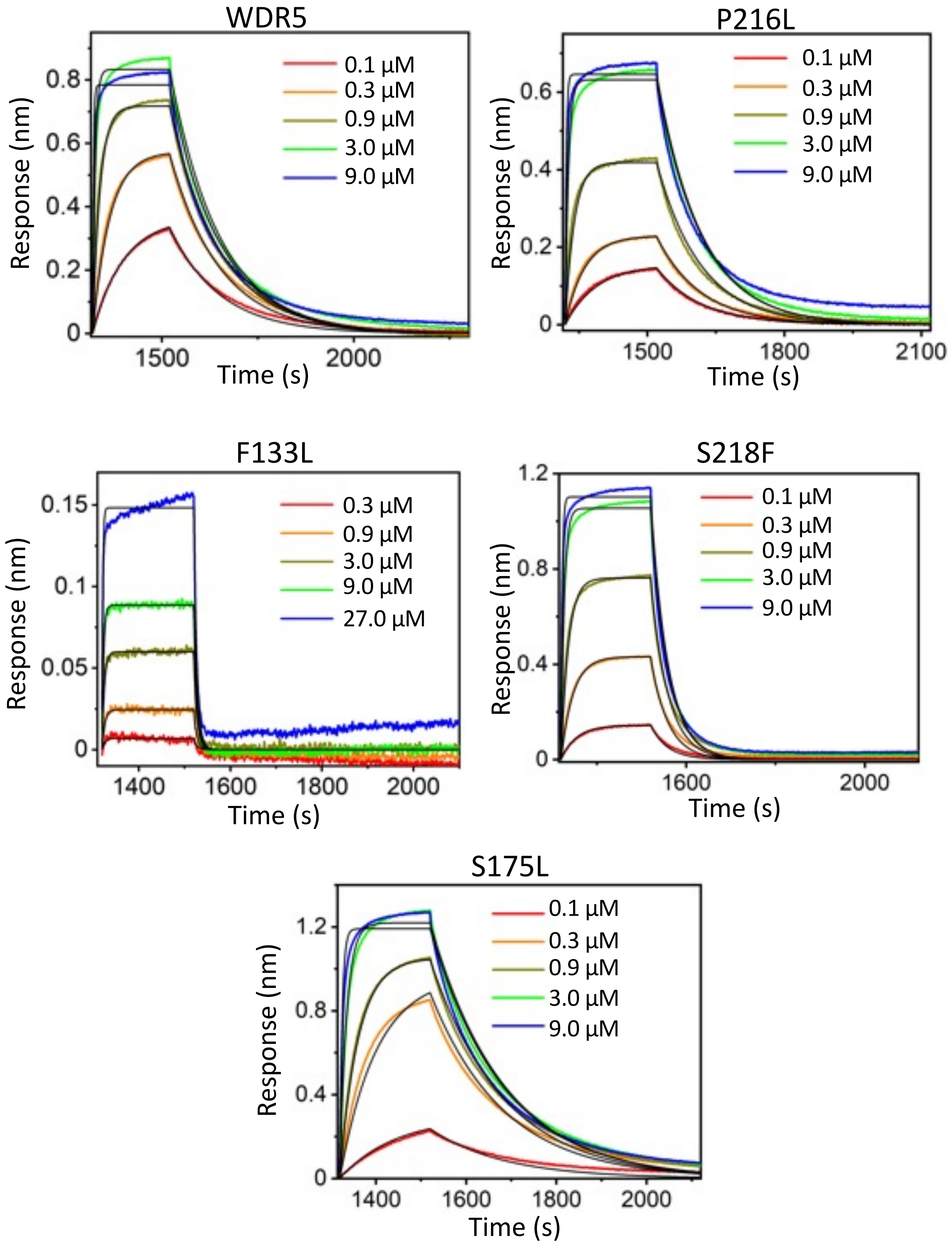

Supplementary Fig. S2. BLI sensorgrams of LT-MLL2$_{\mathbf{Win}}$ interacting with WDR5 and its mutants. 5 nM biotinylated LT-MLL2$_{Win}$ was loaded onto streptavidin-coated sensors for 15 minutes. 3-fold serial dilutions of WDR5 and its mutants were used to obtain individual binding curves. Data for WDR5 is taken from Imran and co-workers (2021).[1] These sensorgrams were fitted to obtain $k_{a\text{-}LT}$, $k_{d\text{-}LT}$, and $K_{D\text{-}LT}$ (**eqs. S1-S3**). The fits are shown in black.

3. Determinations of the kinetic and equilibrium constants of the interactions of ST-SET1$_{Win}$ ligands with WDR5 receptors using BLI measurements.

Supplementary Table S3. Kinetic rate constants of association, $k_{a\text{-}ST}$, of WDR5 and its mutants with ST-SET1$_{Win}$ ligands using BLI measurements. 5 nM biotinylated ST-SET1$_{Win}$ were loaded onto streptavidin-coated sensors for 15 minutes. 3-fold serial dilutions of WDR5 and its mutants, ranging from 0.1 µM to 9 µM, were used to obtain individual binding curves. The buffer solution contained 150 mM NaCl, 20 mM Tris-HCl, 1 mM TCEP, 1 mg/ml bovine serum albumin (BSA), pH 7.5. The binding curves were fitted using the Octet Data Analysis software. $k_{a\text{-}ST}$ values were provided in (M^{-1} s^{-1}) × 10^{-4}. For F133L, 3-fold serial dilutions ranging from 0.3 µM to 27 µM were used. Numbers represent mean ± s.d. determined from three independent BLI experimental determinations.

Peptide	WDR5	P216L	F133L*	S175L**	S218F
MLL2	4.0 ± 0.2	4.2 ± 0.5	1.7 ± 0.1	**3.4 ± 0.3**	4.0 ± 0.2
MLL3	6.6 ± 1.8	4.5 ± 0.3	2.1 ± 0.1	**3.5 ± 0.3**	4.2 ± 0.2
MLL4	2.6 ± 0.3	1.8 ± 0.3	~ 1	**2.0 ± 0.1**	2.1 ± 0.1
SETd1A	6.9 ± 0.4	5.9 ± 0.2	2.7 ± 0.4	**5.7 ± 0.4**	7.4 ± 0.3
SETd1B	4.8 ± 0.1	4.4 ± 0.1	3.0 ± 0.2	**4.8 ± 0.1**	5.3 ± 0.2

*In this case, $k_{a\text{-}ST}$ was in the order of 10^4 $M^{-1}s^{-1}$ assuming that the association process is in the range of values determined with the other MLL peptides. **Experimental values of the test mutant of WDR5.

Supplementary Table S4. Kinetic rate constants of dissociation, $k_{d\text{-}ST}$, of WDR5 and its mutants with ST-MLL ligands using BLI measurements. The N terminus of ST-SET1$_{Win}$ ligands were tagged with biotin and their C-terminus were amidated. 5 nM biotinylated ST-SET1$_{Win}$ ligands were loaded onto streptavidin-coated sensors for 15 minutes. 3-fold serial dilutions of WDR5 and its mutants, ranging from 0.1 µM to 9 µM, were used to obtain individual binding curves. The buffer solution contained 150 mM NaCl, 20 mM Tris-HCl, 1 mM TCEP, 1 mg/ml bovine serum albumin (BSA), pH 7.5. The binding curves were fitted using the Fortebio Octet Data Analysis software. $k_{d\text{-}ST}$ values were provided in (s^{-1}) × 10^3. For F133L, 3-fold serial dilutions ranging from 0.3 µM to 27 µM were used. Numbers represent mean ± s.d. determined from three independent BLI experimental determinations.

Peptide	WDR5	P216L	F133L	S175L**	S218F
MLL2	16 ± 1	21 ± 3	240 ± 10	**12 ± 1**	53 ± 3
MLL3	12 ± 2	23 ± 2	170 ± 10	**28 ± 1**	47 ± 1
MLL4	62 ± 7	350 ± 50	> 1000*	**180 ± 10**	120 ± 10
SETd1A	130 ± 10	79 ± 7	760 ± 120	**160 ± 10**	130 ± 10
SETd1B	29 ± 1	42 ± 3	320 ± 10	**5.4 ± 0.2**	37 ± 3

*This upper-limit value for the detection of $k_{d\text{-}ST}$ is set according to instrument specifications. **Experimental values of the test mutant of WDR5.

<u>Supplementary Table S5.</u> Equilibrium dissociation constants, $K_{D\text{-}ST}$, of WDR5 and its mutants with ST-SET1$_{Win}$ ligands determined from BLI measurements. The N terminus of ST-SET1$_{Win}$ ligands were tagged with biotin and their C-terminus were amidated. 5 nM biotinylated ST-SET1$_{Win}$ ligands were loaded onto streptavidin-coated sensors for 15 minutes. 3-fold serial dilutions of WDR5 and its mutants, ranging from 0.1 µM to 9 µM, were used to obtain individual binding curves. The buffer solution contained 150 mM NaCl, 20 mM Tris-HCl, 1 mM TCEP, 1 mg/ml bovine serum albumin (BSA), pH 7.5. The binding curves were fitted using the Fortebio Octet Data Analysis software. For F133L, 3-fold serial dilutions ranging from 0.3 µM to 27 µM were used. $K_{D\text{-}ST}$ values were provided in nM. Numbers represent mean ± s.d. determined from three independent BLI experimental determinations.

Peptide	WDR5	P216L	F133L	S175L**	S218F
MLL2	410 ± 22	510 ± 50	14,000 ± 2,000	**360 ± 30**	1,300 ± 10
MLL3	190 ± 57	530 ± 30	7,900 ± 400	**810 ± 90**	1,100 ± 100
MLL4	2500 ± 400	20000 ± 5200	≳ 100,000*	**8,500 ± 300**	5,800 ± 500
SETd1A	1800 ± 200	1300 ± 100	29,000 ± 10,000	**2,900 ± 100**	1,700 ± 100
SETd1B	600 ± 30	960 ± 100	10,000 ± 1,000	**110 ± 6**	720 ± 80

*This upper-limit value for the detection of $\boldsymbol{K_{D\text{-}ST}}$ results from dividing the upper-limit value of the detection of $\boldsymbol{k_{d\text{-}ST}}$ by the value of the $\boldsymbol{k_{a\text{-}ST}}$ approximation. **Experimental values of the test mutant of WDR5.

4. The kinetic and equilibrium constants of the interactions of ST-SET1$_{Win}$ ligands with WDR5 receptors normalized to those values corresponding to LT-SET1$_{Win}$ ligands.

<u>Supplementary Table S6.</u> Kinetic rate constant of association, $k_{a\text{-}ST}$, of WDR5 and its mutants, for ST-SET1$_{Win}$ ligands divided by the corresponding $k_{a\text{-}LT}$ for LT-SET1$_{Win}$ ligands. Numbers represent mean ± s.d. determined from three independent BLI experimental determinations.

Peptide	WDR5	P216L	F133L	S175L**	S218F
MLL2	0.89 ± 0.04	0.74 ± 0.10	1.0 ± 0.1	**0.69 ± 0.07**	1.0 ± 0.1
MLL3	1.2 ± 0.4	0.84 ± 0.08	0.59 ± 0.02	**0.71 ± 0.08**	1.0 ± 0.1
MLL4	1.1 ± 0.2	0.96 ± 0.17	ND*	**0.68 ± 0.03**	1.1 ± 0.1
SETd1A	0.83 ± 0.06	0.69 ± 0.02	1.07 ± 0.19	**0.93 ± 0.07**	1.3 ± 0.1
SETd1B	0.68 ± 0.01	0.55 ± 0.02	0.70 ± 0.05	**0.57 ± 0.01**	0.85 ± 0.04

*ND stands for "<u>N</u>ot <u>D</u>etermined." Interaction between F133L and MLL4 was detectable using a BLI measurement. However, no statistically significant accurate determinations were made due to limited resolution of the approach. **Experimental values of the test mutant of WDR5.

<u>Supplementary Table S7.</u> Kinetic rate constant of disassociation, $k_{d\text{-}ST}$, of WDR5 and its mutants interacting with ST-SET1$_{Win}$ ligands divided by the corresponding $k_{d\text{-}LT}$ for LT-SET1$_{Win}$ ligands. Numbers represent mean ± s.d. determined from three independent BLI experimental determinations.

Peptide	WDR5	P216L	F133L	S175L**	S218F
MLL2	2.1 ± 0.1	1.9 ± 0.4	1.7 ± 0.1	**2.0 ± 0.1**	2.2 ± 0.1
MLL3	2.2 ± 0.4	2.1 ± 0.2	1.6 ± 0.1	**1.7 ± 0.1**	2.2 ± 0.1
MLL4	1.6 ± 0.2	1.6 ± 0.2	ND*	**1.4 ± 0.1**	1.7 ± 0.1
SETd1A	2.5 ± 0.3	2.4 ± 0.3	1.1 ± 0.2	**1.8 ± 0.1**	2.7 ± 0.2
SETd1B	1.7 ± 0.1	1.4 ± 0.1	1.3 ± 0.1	**1.1 ± 0.1**	1.6 ± 0.1

*ND stands for "Not Determined." Interaction between F133L and MLL4 was detectable using a BLI measurement. However, no statistically significant accurate determinations were made due to limited resolution of the approach. **Experimental values of the test mutant of WDR5.

<u>Supplementary Table S8.</u> Calculations of the differential activation free energies of the dissociation processes, $\Delta\Delta G_d$, of the interactions of ST-SET1$_{Win}$ ligands with respect to those of LT-SET1$_{Win}$ ligands. Calculated values of $\Delta\Delta G_d$ are given in kcal/mol. Data are provided as mean ± s.d. from three independent BLI experimental determinations.

Peptide	WDR5	P216L	F133L	S175L**	S218F
MLL2	-0.45 ± 0.01	-0.38 ± 0.12	-0.33 ± 0.04	**-0.40 ± 0.02**	-0.45 ± 0.04
MLL3	-0.45 ± 0.10	-0.43 ± 0.07	-0.30 ± 0.03	**-0.32 ± 0.02**	-0.47 ± 0.01
MLL4	-0.28 ± 0.08	-0.28 ± 0.10	ND*	**-0.19 ± 0.01**	-0.33 ± 0.04
SETd1A	-0.53 ± 0.06	-1.88 ± 0.06	-0.04 ± 0.11	**-0.36 ± 0.03**	-0.59 ± 0.04
SETd1B	-0.30 ± 0.03	-0.21 ± 0.06	-0.16 ± 0.02	**-0.06 ± 0.03**	-0.28 ± 0.05

*ND stands for "Not Determined." Interaction between F133L and MLL4 was detectable using a BLI measurement. However, no quantitative determinations were made due to limited time resolution of the approach. **Experimental values of the test mutant of WDR5.

Supplementary Table S9. $K_{D\text{-}ST}$ measured with ST-SET1$_{Win}$ ligands normalized to the corresponding $K_{D\text{-}LT}$ values measured with LT-SET1$_{Win}$ ligands. Numbers represent mean ± s.d. determined from three independent BLI experimental determinations.

Peptide	WDR5	P216L	F133L	S175L**	S218F
MLL2	2.4 ± 0.2	2.5 ± 0.3	1.7 ± 0.2	**2.8 ± 0.3**	2.1 ± 0.1
MLL3	1.9 ± 0.7	2.6 ± 0.2	2.1 ± 0.1	**2.5 ± 0.3**	2.2 ± 0.2
MLL4	1.5 ± 0.3	2.0 ± 0.6	ND*	**2.0 ± 0.1**	1.7 ± 0.2
SETd1A	3.0 ± 0.4	3.5 ± 0.4	0.96 ± 0.39	**2.0 ± 0.1**	2.0 ± 0.1
SETd1B	2.4 ± 0.1	2.6 ± 0.3	1.7 ± 0.1	**2.0 ± 0.1**	2.0 ± 0.3

*ND stands for "Not Determined." Interaction between F133L and MLL4 was detectable using a BLI measurement. However, no quantitative determinations were made due to limited time resolution of the approach. **Experimental values of the test mutant of WDR5.

Supplementary Table S10. Calculations of the differential activation free energies of the ligand-receptor complex formation, $\Delta\Delta G$, of WDR5-SET1$_{Win}$ interactions of ST-SET1$_{Win}$ ligands with respect to those of LT-SET1$_{Win}$ ligands. Calculated values of $\Delta\Delta G$ are given in kcal/mol. Data are provided as mean ± s.d. from three independent BLI experimental determinations.

Peptide	WDR5	P216L	F133L	S175L**	S218F
MLL2	0.51 ± 0.04	0.54 ± 0.07	0.32 ± 0.08	**0.61 ± 0.06**	0.43 ± 0.01
MLL3	0.37 ± 0.21	0.57 ± 0.05	0.45 ± 0.04	**0.53 ± 0.08**	0.45 ± 0.05
MLL4	0.22 ± 0.13	0.40 ± 0.20	ND*	**0.42 ± 0.02**	0.30 ± 0.06
SETd1A	0.64 ± 0.07	0.74 ± 0.07	-0.05 ± 0.22	**0.42 ± 0.02**	0.42 ± 0.03
SETd1B	0.53 ± 0.03	0.57 ± 0.08	0.32 ± 0.05	**0.40 ± 0.04**	0.40 ± 0.08

*ND stands for "Not Determined." Interaction between F133L and MLL4 was detectable using a BLI measurement. However, no quantitative determinations were made due to limited time resolution of the approach. **Experimental values of the test mutant of WDR5.

5. Scatter plots of the association rate constants versus the dissociation rate constants using linear- and logarithm-scale representations.

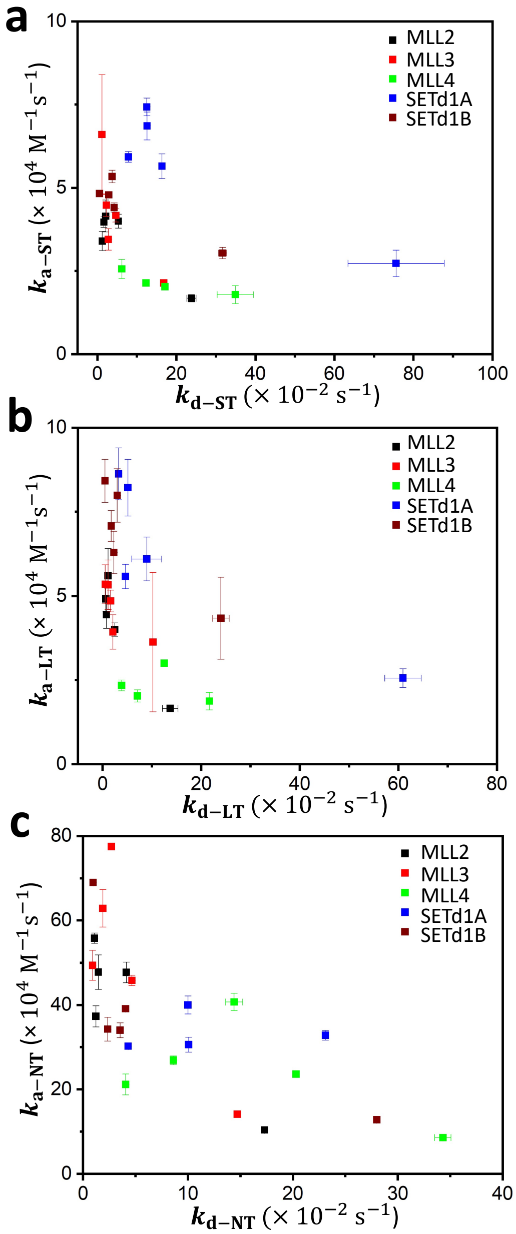

<u>Supplementary Fig. S3.</u> Scatter plots of the association rate constants versus the dissociation rate constants using a linear-scale representation. (a) Data resulted from short-tether (ST) experiments. **(b)** Data resulted from long-tether (LT) experiments. **(c)** Data resulted from no tether (NT) experiments. For ST and LT experiments, $MLL4_{Win}$-F133L interactions were not quantitatively determined. Hence, they only have four points each for MLL4 (for WDR5, P216L, S218F and S175L). For NT experiments, $SETd1A_{Win}$-F133L interactions were not quantitatively determined. Therefore, they only have four points for SETd1A (for WDR5, P216L, S218F and S175L). Data are provided as mean ± s.d. from three independent experimental determinations.

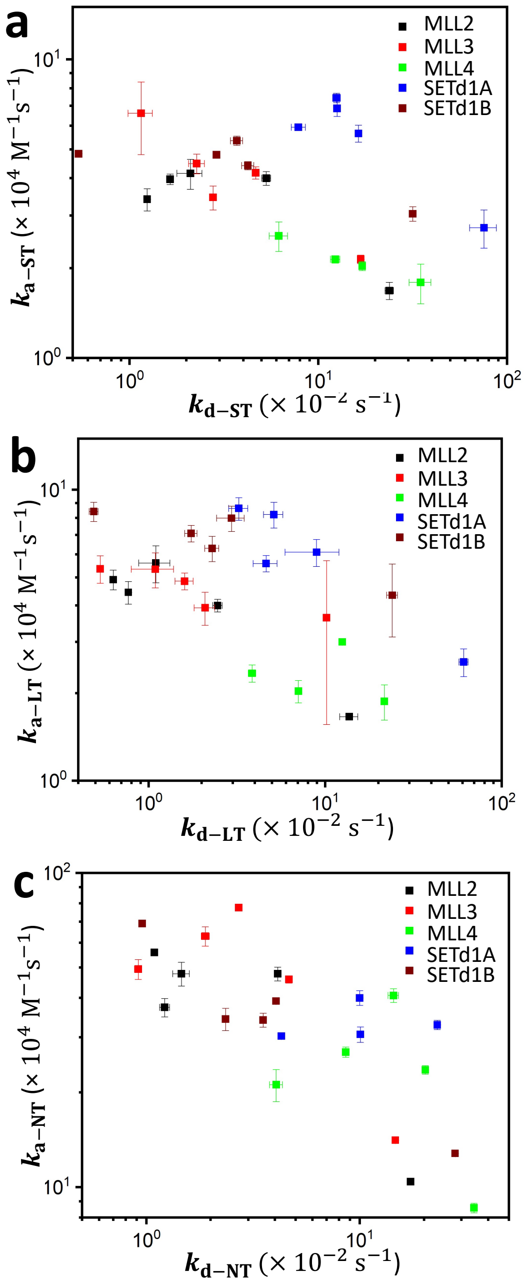

Supplementary Fig. S4. Scatter plots of the association rate constants versus the dissociation rate constants using a logarithm-scale representation. (a) Data resulted from short-tether (ST) experiments. **(b)** Data resulted from long-tether (LT) experiments. **(c)** Data resulted from no tether (NT; SPR) experiments. For ST and LT experiments, $MLL4_{Win}$-F133L interactions were not quantitatively determined. Hence, they only have four points each for MLL4 (for WDR5, P216L, S218F and S175L). For NT experiments, $SETd1A_{Win}$-F133L interactions were not quantitatively determined. Therefore, they only have four points for SETd1A (for WDR5, P216L, S218F and S175L). Data are provided as mean ± s.d. from three independent experimental determinations.

6. Examples of SPR sensorgrams and fittings for probing the real-time kinetics of NT-SET1$_{Win}$-WDR5 interactions.

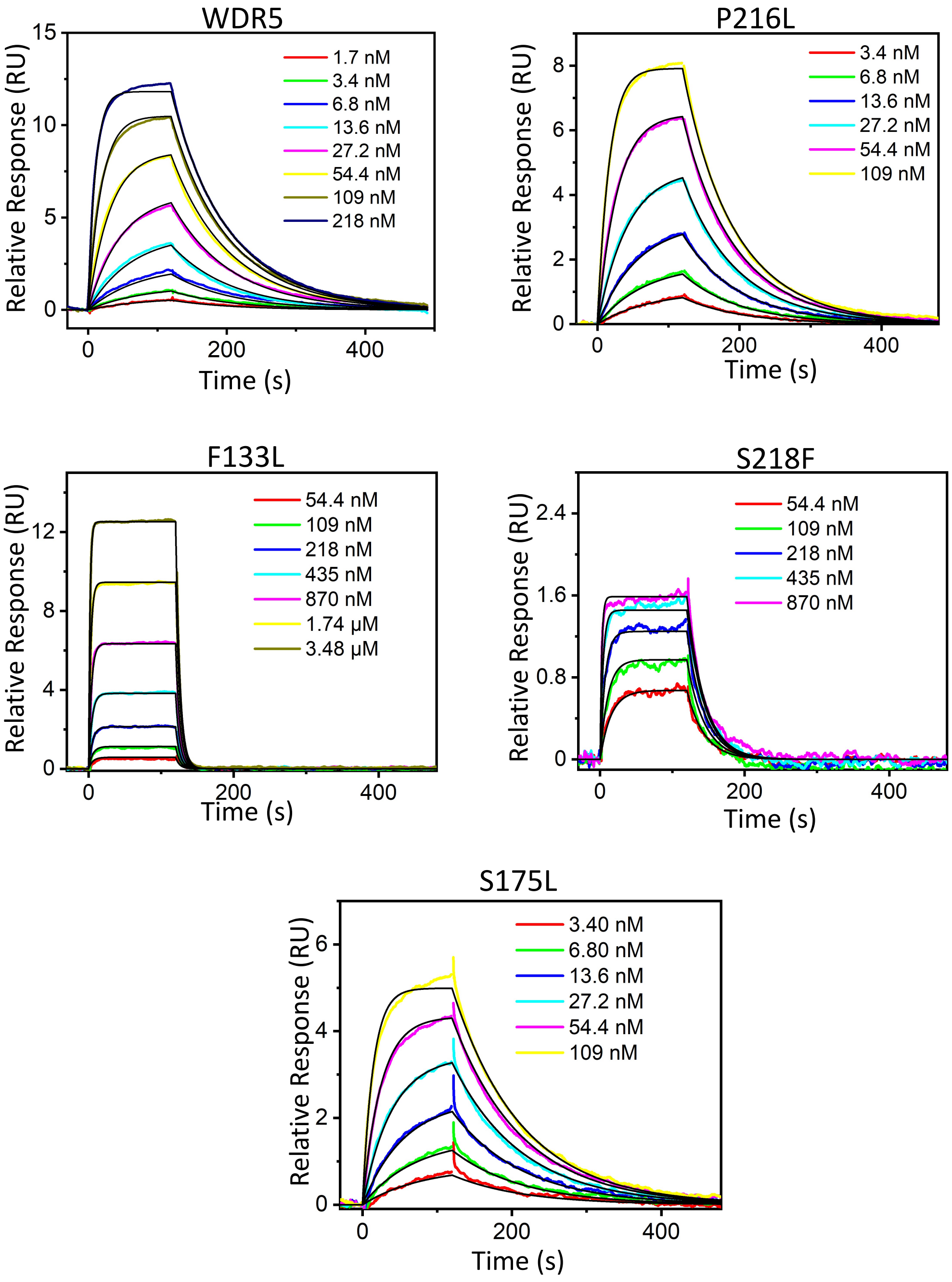

<u>Supplementary Fig. S5.</u> SPR sensorgrams of NT-MLL2$_{Win}$ interacting with immobilized WDR5 proteins. WDR5 and its mutants were immobilized onto Cytiva Series S CM5 chips using EDC/NHS amine coupling chemistry in separate experiments. Titration series of no-tether MLL2$_{Win}$ (NT-MLL2$_{Win}$) was injected as analyte and the corresponding association (120 sec.) and dissociation (360 sec.) curves are shown. Data for WDR5 is taken from Imran and co-workers (2021).[1] These sensorgrams were fitted to obtain $k_{a\text{-NT}}$, $k_{d\text{-NT}}$, and $K_{D\text{-NT}}$ (**eqs. S1-S3**). The fits are shown in black.

7. Determinations of the kinetic and equilibrium constants of the interactions of NT-SET1$_{Win}$ ligands with WDR5 receptors using SPR measurements.

<u>Supplementary Table S11.</u> Kinetic rate constants of association, $k_{a\text{-}NT}$, of immobilized WDR5 receptor and its mutants with NT-SET1$_{Win}$ ligands using SPR measurements. WDR5 and its mutants were immobilized onto Cytiva Series S CM5 chips using EDC/NHS amine coupling chemistry. Titration series of the respective NT-SET1$_{Win}$ ligands were injected as analytes. In the case of the SETd1A-F133L binding interaction, the kinetic constants were outside the limits that could be measured by the instrument. $k_{a\text{-}NT}$ values were provided in ($M^{-1}s^{-1}$) × 10^{-4}. Values represent mean ± s.d. acquired from three independent SPR experimental determinations.

Peptide	WDR5*	P216L	F133L	S175L***	S218F
MLL2	37 ± 3	48 ± 4	10 ± 1	**56 ± 1**	48 ± 2
MLL3	49 ± 4	63 ± 4	14 ± 1	**78 ± 1**	46 ± 1
MLL4	21 ± 2	24 ± 1	8.6 ± 0.3	**41 ± 2**	27 ± 1
SETd1A	31 ± 2	30 ± 1	~ 10**	**33 ± 1**	40 ± 2
SETd1B	34 ± 3	34 ± 2	13 ± 1	**69 ± 1**	39 ± 1

*Data from Imran and co-workers.[1] **Interaction between wild-type F133L and SETd1A was detectable using a SPR measurement. However, no quantitative determinations were made due to limited time resolution of the approach. In this case, $k_{a\text{-}NT}$ was in the order of 10^5 $M^{-1}s^{-1}$ assuming that the association process is in the range of values determined with the other NT-SET1$_{Win}$ ligands. ***The test mutant of WDR5.

<u>Supplementary Table S12.</u> Kinetic rate constants of dissociation, $k_{d\text{-}NT}$, of WDR5 and its mutants with the NT-SET1$_{Win}$ ligands using SPR measurements. WDR5 proteins were immobilized onto Cytiva Series S CM5 chips using EDC/NHS amine coupling chemistry. Titration series of the respective SET1$_{Win}$ peptide ligands were injected as analytes. In the case of the SETd1A-F133L binding interaction, the kinetic constants were outside the limits that could be measured by the instrument. $k_{d\text{-}NT}$ values were provided in (s^{-1}) × 10^3. Values represent mean ± s.d. acquired from three independent SPR experimental determinations.

Peptide	WDR5*	P216L	F133L	S175L***	S218F
MLL2	12 ± 1	15 ± 1	170 ± 10	**11 ± 1**	41 ± 1
MLL3	9.2 ± 0.1	19 ± 1	150 ± 10	**27 ± 1**	47 ± 1
MLL4	41 ± 3	200 ± 10	340 ± 10	**140 ± 10**	86 ± 2
SETd1A	110 ± 10	43 ± 1	> 500**	**230 ± 10**	100 ± 10
SETd1B	24 ± 1	35 ± 1	280 ± 10	**9.6 ± 0.3**	41 ± 1

*Data from Imran and co-workers.[1] **The upper-limit value for the detection of $k_{d\text{-}NT}$ using SPR experiments is explicitly specified by the instrument manufacturer. The Biacore 8K+ cannot measure rate constants of dissociation, $k_{d\text{-}NT}$, faster than 0.5 s^{-1}. ***The test mutant of WDR5.

Supplementary Table S13. Equilibrium dissociation constants, $K_{D\text{-}NT}$, of WDR5 and its mutants with the NT-SET1$_{Win}$ ligands using SPR measurements. Either WDR5 or its derivatives was immobilized onto Cytiva Series S CM5 chips using EDC/NHS amine coupling chemistry. Titration series of the respective NT-SET1$_{Win}$ ligands were injected as analytes. $K_{D\text{-}NT}$ was calculated directly from these kinetic rate constants using $K_D = k_d/k_a$. In the case of the SETd1A-F133L binding interaction, the kinetic constants were outside the limits that could be measured by the instrument. Therefore, an affinity analysis (relative response vs. concentration dose-response curve) was used to calculate the $K_{D\text{-}NT}$. $K_{D\text{-}NT}$ values were provided in nM. Values represent mean ± s.d. acquired from three independent SPR experimental determinations.

Peptide	WDR5*	P216L	F133L	S175L***	S218F
MLL2	33 ± 2	31 ± 1	1,700 ± 100	**20 ± 1**	87 ± 5
MLL3	19 ± 1	30 ± 1	1,000 ± 100	**35 ± 1**	100 ± 10
MLL4	190 ± 20	860 ± 20	4,000 ± 100	**350 ± 10**	320 ± 10
SETd1A	350 ± 10	140 ± 10	11,000 ± 1,000**	**710 ± 20**	250 ± 10
SETd1B	69 ± 6	110 ± 10	2,200 ± 100	**14 ± 1**	100 ± 10

*Data from Imran and co-workers.[1] **Here, $K_{D\text{-}NT}$ was determined using a steady-state SPR measurement. ***The test mutant of WDR5.

8. The kinetic and equilibrium constants of the interactions of NT-SET1$_{Win}$ ligands with WDR5 receptors normalized to the corresponding of LT-SET1$_{Win}$ ligands.

Supplementary Table S14. Kinetic rate constant of association, $k_{a\text{-}NT}$, of WDR5 and its mutants, determined by SPR measurements divided by the corresponding $k_{a\text{-}LT}$ determined by BLI sensorgrams. Data are provided as mean ± s.d. from three independent experiments.

Peptide	WDR5	P216L	F133L	S175L***	S218F
MLL2	8.4 ± 0.7	8.5 ± 0.7	6.2 ± 0.1	**11 ± 1**	12 ± 1
MLL3	9.2 ± 0.7	12 ± 1	3.9 ± 0.1	**16 ± 1**	12 ± 1
MLL4	9.0 ± 1.1	12 ± 1	ND*	**14 ± 1**	13 ± 1
SETd1A	3.7 ± 0.2	3.5 ± 0.1	ND**	**5.4 ± 0.2**	7.2 ± 0.4
SETd1B	4.8 ± 0.4	4.3 ± 0.2	3.0 ± 0.1	**8.2 ± 0.1**	6.2 ± 0.1

*ND stands for "Not Determined." Interaction between F133L and MLL4 was detectable using a BLI measurement. However, this interaction was not quantitatively determined using BLI due to the limited time resolution of the approach. ** Interaction between F133L and SETd1A was detectable using a SPR measurement. However, this interaction was not quantitatively determined using SPR due to the limited resolution of the approach. ***The test mutant of WDR5.

<u>Supplementary Table S15.</u> Kinetic rate constants of dissociation, $k_{d\text{-}NT}$, of WDR5 and its mutants, determined by SPR, normalized to the corresponding $k_{d\text{-}LT}$ values. Data are provided as mean ± s.d. from three independent experiments.

Peptide	WDR5	P216L	F133L	S175L***	S218F
MLL2	1.6 ± 0.1	1.3 ± 0.1	1.3 ± 0.1	**1.7 ± 0.1**	1.7 ± 0.1
MLL3	1.7 ± 0.1	1.7 ± 0.1	1.4 ± 0.1	**1.7 ± 0.1**	2.2 ± 0.1
MLL4	1.1 ± 0.1	0.94 ± 0.01	ND*	**1.2 ± 0.1**	1.2 ± 0.1
SETd1A	2.1 ± 0.1	1.3 ± 0.1	ND**	**2.6 ± 0.1**	2.2 ± 0.1
SETd1B	1.3 ± 0.1	1.2 ± 0.1	1.2 ± 0.1	**2.0 ± 0.1**	1.8 ± 0.1

*ND stands for "Not Determined." **The interaction between F133L and SETd1A was detectable using an SPR measurement. Yet, this interaction was not quantitatively determined using BLI due to the limited time resolution of the approach. ***The test mutant of WDR5.

<u>Supplementary Table S16.</u> $K_{D\text{-}NT}$ values determined by SPR measurements, which were normalized to the corresponding $K_{D\text{-}LT}$ values determined by BLI measurements. Data are provided as mean ± s.d. from three independent experiments.

Peptide	WDR5	P216L	F133L	S175L**	S218F
MLL2	0.19 ± 0.01	0.15 ± 0.01	0.20 ± 0.01	**0.15 ± 0.01**	0.13 ± 0.01
MLL3	0.19 ± 0.01	0.15 ± 0.01	0.28 ± 0.01	**0.11 ± 0.01**	0.19 ± 0.01
MLL4	0.12 ± 0.01	0.087 ± 0.002	ND*	**0.085 ± 0.001**	0.093 ± 0.002
SETd1A	0.56 ± 0.02	0.38 ± 0.01	0.37 ± 0.01	**0.49 ± 0.02**	0.30 ± 0.02
SETd1B	0.27 ± 0.02	0.29 ± 0.01	0.36 ± 0.01	**0.25 ± 0.01**	0.28 ± 0.01

ND stands for "Not Determined." The interaction between F133L and MLL4 was detectable, but not quantifiable using BLI. **The test mutant of WDR5.

<u>Supplementary Table S17.</u> Differential activation free energies of the ligand-receptor complex formation, $\Delta\Delta G$ (kcal/mol), determined for NT conditions with respect to LT conditions. Data are provided as mean ± s.d. using three independent experiments.

Peptide	WDR5	P216L	F133L	S175L**	S218F
MLL2	-0.98 ± 0.03	-1.1 ± 0.1	-0.94 ± 0.01	**-1.1 ± 0.1**	-1.2 ± 0.1
MLL3	-0.99 ± 0.02	-1.1 ± 0.1	-0.75 ± 0.01	**-1.3 ± 0.1**	-0.97 ± 0.02
MLL4	-1.3 ± 0.1	-1.4 ± 0.1	ND*	**-1.5 ± 0.1**	-1.4 ± 0.1
SETd1A	-0.34 ± 0.02	-0.57 ± 0.01	-0.58 ± 0.01	**-0.42 ± 0.02**	-0.70 ± 0.03
SETd1B	-0.76 ± 0.05	-0.73 ± 0.02	-0.60 ± 0.01	**-0.83 ± 0.02**	-0.74 ± 0.01

*ND stands for "Not Determined." The interaction between F133L and MLL4 was detectable, but not quantifiable using BLI. **The test mutant of WDR5.

9. The 3D plots and contour maps of the association rate constants under ST and LT conditions normalized to those recorded under NT conditions.

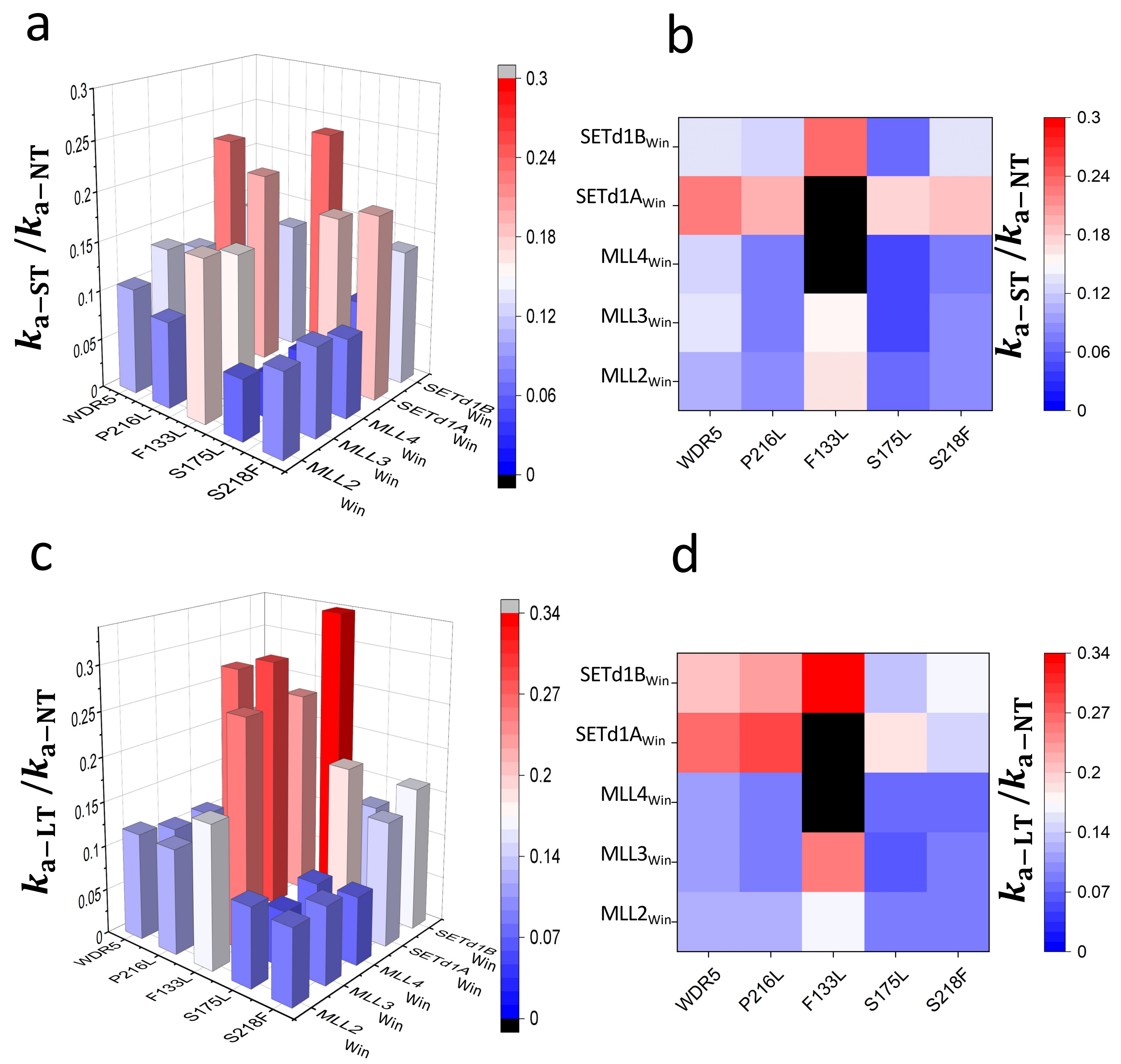

<u>Supplementary Fig. S6.</u> 3D plots and contour maps of the normalized association rate constants. **(a)** Bar graph and **(b)** contour map of $k_{a\text{-}ST}$ values for the interaction of ST-SET1$_{Win}$ ligands, with WDR5 and its mutants, divided by their corresponding $k_{a\text{-}NT}$ values. **(c)** Bar graph and **(d)** contour map of $k_{a\text{-}LT}$ values for the interaction of LT-SET1$_{Win}$ ligands, with WDR5 and its mutants, divided by their corresponding $k_{a\text{-}NT}$ values. $k_{a\text{-}ST}$ for the MLL4$_{Win}$-F133L interactions could not be determined using BLI, while $k_{a\text{-}NT}$ of the SETd1A$_{Win}$-F133L interactions could not be determined using SPR. Therefore, those values are colored in black.

10. The 3D plots and contour maps of the dissociation rate constants under ST and LT conditions normalized to those recorded under NT conditions.

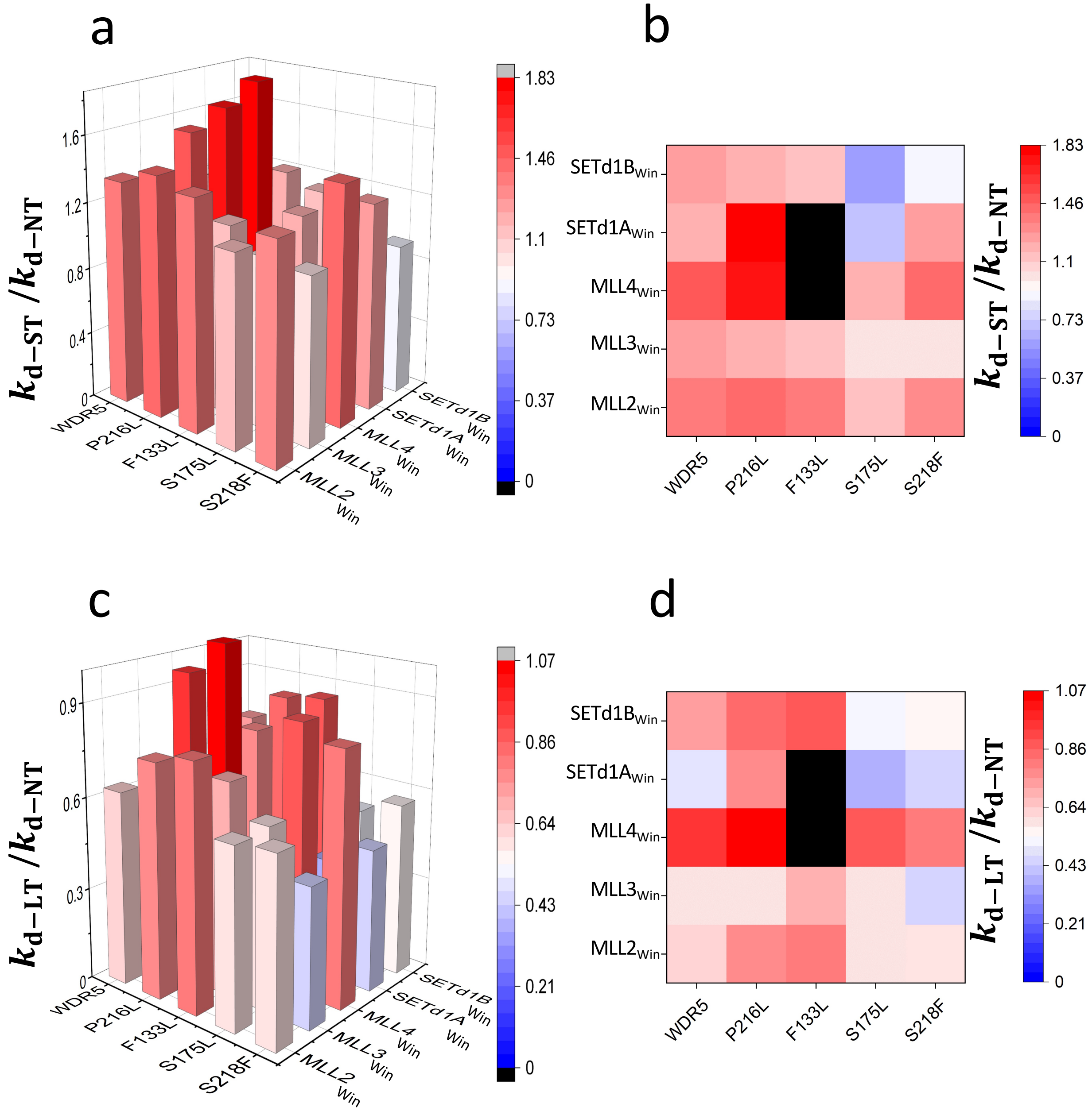

<u>Supplementary Fig. S7.</u> 3D plots and contour maps of the normalized association rate constants. (a) Bar graph and **(b)** contour map of $k_{d\text{-ST}}$ values for the interaction of ST-SET1$_{Win}$ ligands, with WDR5 and its mutants, divided by their corresponding $k_{d\text{-NT}}$ values. (c) Bar graph and **(d)** contour map of $k_{d\text{-LT}}$ values for the interaction of LT-SET1$_{Win}$ ligands, with WDR5 and its mutants, divided by their corresponding $k_{d\text{-NT}}$ values. $k_{d\text{-ST}}$ for the MLL4$_{Win}$-F133L interactions could not be determined using BLI, while $k_{d\text{-NT}}$ of the SETd1A$_{Win}$-F133L interactions could not be determined using SPR. Therefore, those values are colored in black.

11. Scatter plots of kinetic and equilibrium constants for the ST, LT, and NT experiments.

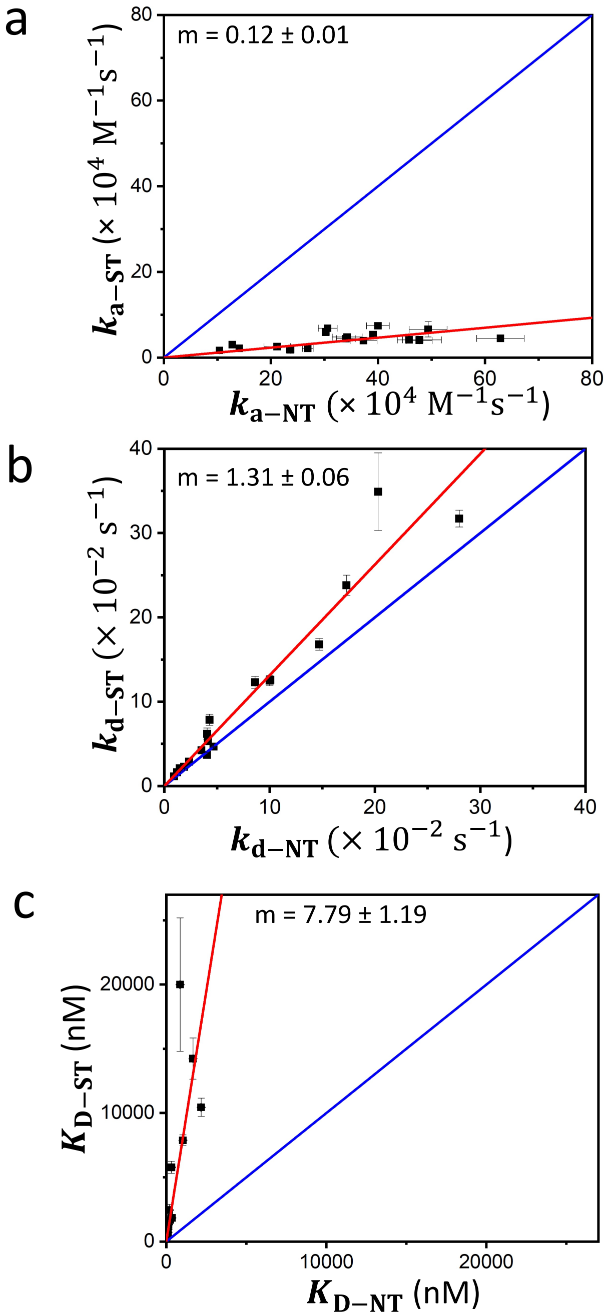

Supplementary Fig. S8. Scatter plots of the k_a, k_d, and K_D for the ST and NT conditions. **(a)** $k_{a\text{-ST}}$ values plotted against the corresponding $k_{a\text{-NT}}$ values. Points below this line correspond to interactions with slower association rates for the ST experiments. **(b)** $k_{d\text{-ST}}$ values plotted against the corresponding $k_{d\text{-NT}}$ values. Points above this line correspond to interactions with faster disassociation rate constants for the ST experiments. **(c)** $K_{D\text{-ST}}$ values plotted against the corresponding $K_{D\text{-NT}}$ values. Points above this line correspond to interactions that were weaker in the ST experiments. Data are provided as mean ± s.d. from three independent experiments.

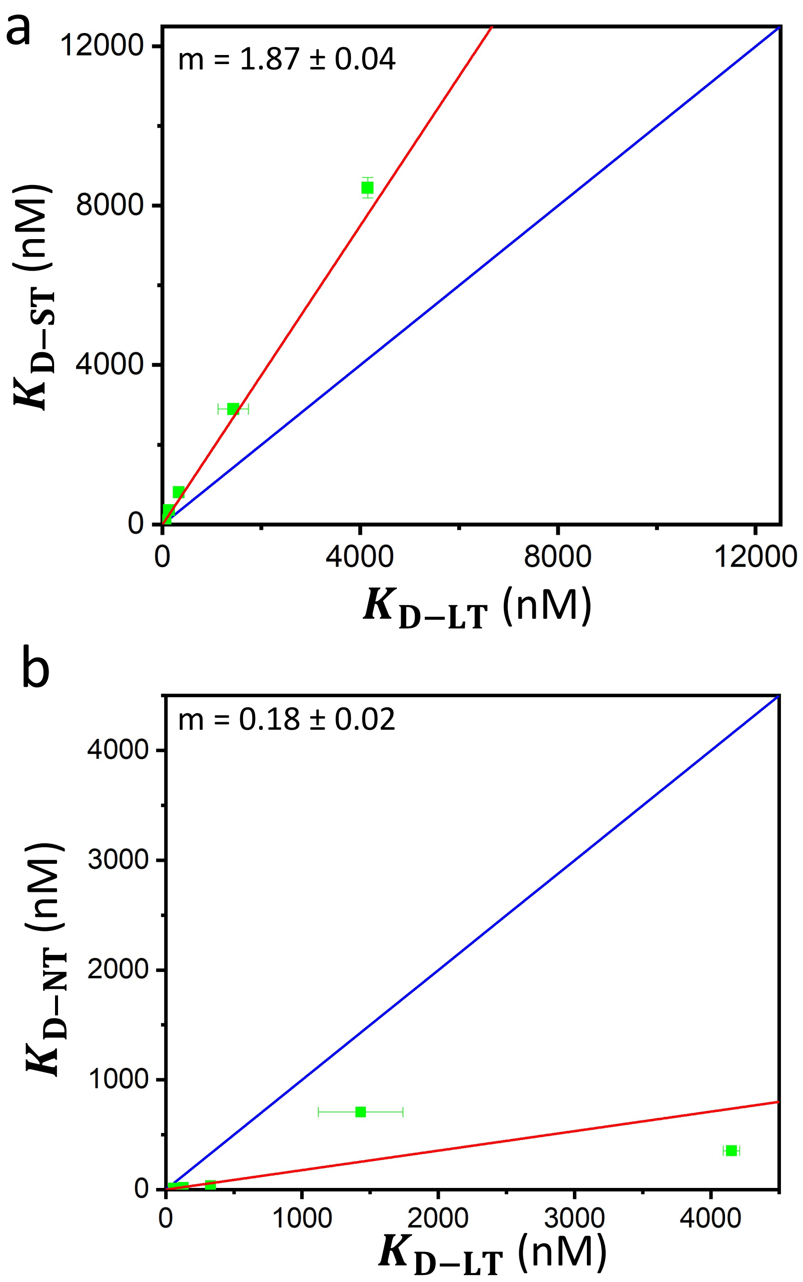

Supplementary Fig. S9. Scatter plots of the equilibrium dissociation constants of S175L data. (a) $K_{D\text{-}ST}$ values plotted against the $K_{D\text{-}LT}$ values. The red line is the fit resulted from interactions of WDR5, P216L, S218F and F133L with $SET1_{Win}$ under ST and LT conditions. The green points represent interactions of S175L. Points above this blue line correspond to interactions which were weakened by reducing tether length. **(b)** $K_{D\text{-}NT}$ values plotted against the $K_{D\text{-}LT}$ values. The red line is the fit resulted from interactions of WDR5, P216L, S218F and F133L with $SET1_{Win}$ under NT and LT conditions. The green points represent interactions of S175L. Points below this blue line correspond to interactions that were stronger under NT conditions. m indicates the slope of curves in both panels. Data are provided as mean ± s.d. from three independent experiments.

12. Supporting references.

(1) Imran, A.; Moyer, B. S.; Canning, A. J.; Kalina, D.; Duncan, T. M.; Moody, K. J.; Wolfe, A. J.; Cosgrove, M. S.; Movileanu, L. Kinetics of the multitasking high-affinity Win binding site of WDR5 in restricted and unrestricted conditions. *Biochem. J.* **2021**, *478*, 2145-2161.

(2) Patel, A.; Dharmarajan, V.; Cosgrove, M. S. Structure of WDR5 bound to mixed lineage leukemia protein-1 peptide. *J. Biol. Chem.* **2008**, *283*, 32158-32161.

(3) Patel, A.; Vought, V. E.; Dharmarajan, V.; Cosgrove, M. S. A conserved arginine-containing motif crucial for the assembly and enzymatic activity of the mixed lineage leukemia protein-1 core complex. *J. Biol. Chem.* **2008**, *283*, 32162-32175.

(4) Tate, J. G.; Bamford, S.; Jubb, H. C.; Sondka, Z.; Beare, D. M.; Bindal, N.; Boutselakis, H.; Cole, C. G.; Creatore, C.; Dawson, E.; Fish, P.; Harsha, B.; Hathaway, C.; Jupe, S. C.; Kok, C. Y.; Noble, K.; Ponting, L.; Ramshaw, C. C.; Rye, C. E.; Speedy, H. E.; Stefancsik, R.; Thompson, S. L.; Wang, S.; Ward, S.; Campbell, P. J.; Forbes, S. A. COSMIC: the Catalogue Of Somatic Mutations In Cancer. *Nucleic Acids Res.* **2019**, *47*, D941-d947.

(5) Forbes, S. A.; Beare, D.; Boutselakis, H.; Bamford, S.; Bindal, N.; Tate, J.; Cole, C. G.; Ward, S.; Dawson, E.; Ponting, L.; Stefancsik, R.; Harsha, B.; Kok, C. Y.; Jia, M.; Jubb, H.; Sondka, Z.; Thompson, S.; De, T.; Campbell, P. J. COSMIC: somatic cancer genetics at high-resolution. *Nucleic Acids Res.* **2017**, *45*, D777-d783.

(6) Goldman, M. J.; Zhang, J.; Fonseca, N. A.; Cortés-Ciriano, I.; Xiang, Q.; Craft, B.; Piñeiro-Yáñez, E.; O'Connor, B. D.; Bazant, W.; Barrera, E.; Muñoz-Pomer, A.; Petryszak, R.; Füllgrabe, A.; Al-Shahrour, F.; Keays, M.; Haussler, D.; Weinstein, J. N.; Huber, W.; Valencia, A.; Park, P. J.; Papatheodorou, I.; Zhu, J.; Ferretti, V.; Vazquez, M. A user guide for the online exploration and visualization of PCAWG data. *Nat. Commun.* **2020**, *11*, 3400.

(7) Weeramange, C. J.; Fairlamb, M. S.; Singh, D.; Fenton, A. W.; Swint-Kruse, L. The strengths and limitations of using biolayer interferometry to monitor equilibrium titrations of biomolecules. *Protein Sci.* **2020**, *29*, 1018-1034.

(8) Dharmarajan, V.; Lee, J. H.; Patel, A.; Skalnik, D. G.; Cosgrove, M. S. Structural basis for WDR5 interaction (Win) motif recognition in human SET1 family histone methyltransferases. *J. Biol. Chem.* **2012**, *287*, 27275-27289.

(9) Zhang, P.; Lee, H.; Brunzelle, J. S.; Couture, J. F. The plasticity of WDR5 peptide-binding cleft enables the binding of the SET1 family of histone methyltransferases. *Nucleic Acids Res.* **2012**, *40*, 4237-4246.

(10) Movileanu, L.; Cheley, S.; Howorka, S.; Braha, O.; Bayley, H. Location of a Constriction in the Lumen of a Transmembrane Pore by Targeted Covalent Attachment of Polymer Molecules. *J. Gen. Physiol.* **2001**, *117*, 239-251.

(11) Wolfe, A. J.; Gugel, J. F.; Chen, M.; Movileanu, L. Kinetics of Membrane Protein-Detergent Interactions Depend on Protein Electrostatics. *J. Phys. Chem. B* **2018**, *122*, 9471-9481.

(12) Jarmoskaite, I.; AlSadhan, I.; Vaidyanathan, P. P.; Herschlag, D. How to measure and evaluate binding affinities. *eLife* **2020**, *9*, e57264.
